# Divergent Gut Microbiota Configurations Are Associated with Contrasting Physiological Profiles in Grazing Yaks and Introduced Feedlot Holstein Cattle Under High-Altitude Conditions

**DOI:** 10.3390/microorganisms14081647

**Published:** 2026-07-28

**Authors:** Ding Li, Haozheng Li, Shu Zhang, Tong Wu, Dongyang Ye, Yaping Jin

**Affiliations:** 1 College of Veterinary Medicine, Northwest A&F University, Yangling 712100, China; 15891740706@163.com (D.L.);; 2 College of Animal Science and Technology, Xizang Vocational Technical College, Lhasa 850000, China; 3 Key Laboratory of Ruminant Disease Prevention and Control, Ministry of Agriculture and Rural Affairs, Yangling 712100, China

**Keywords:** gut microbiota, high-altitude adaptation, yak (*Bos grunniens*), Holstein cattle, metagenomics, immune modulation, fiber digestibility

## Abstract

The Qinghai–Tibet Plateau presents extreme environmental challenges, including hypobaric hypoxia and nutritional scarcity, to which indigenous yaks (*Bos grunniens*) are remarkably adapted, whereas introduced Holstein cattle (*Bos taurus*) often exhibit severe maladaptation. This study compared the gut microbiota of grazing yaks (*n* = 20) and feedlot Holstein cattle (*n* = 20) under high-altitude conditions using 16S rRNA gene amplicon sequencing (for community composition) and shotgun metagenomic sequencing (for direct functional profiling of CAZy, COG, and KEGG pathways), combined with in vitro fermentation, digestive enzyme assays, and serum immune- and growth-related indicators. Results: The yak gut microbiota was dominated by Proteobacteria (35 ± 4%) and Actinobacteria (15 ± 2%), whereas Holstein cattle were dominated by *Firmicutes* (40 ± 4%) and *Bacteroidetes* (25 ± 3%). At the species level, NR-based metagenomic annotation suggested that Acinetobacter-related taxa, putatively assigned as Acinetobacter pseudolwoffii and A. lwoffii, were abundant in yaks. Functional profiling revealed that the yak microbiota was enriched in CAZy families CE1, GT2, and GT4, whereas the feedlot Holstein cattle microbiota showed enrichment of several KEGG orthologs related to multidrug efflux and carbohydrate transport, including dinF/mepA/vmrA and susC/susD. In vitro fermentation using enriched culturable fecal bacterial consortia showed higher dry matter digestibility and digestive enzyme activities in yak-derived consortia than in Holstein-derived consortia. For serum immune indicators, IgG was significantly higher in feedlot Holstein cattle, whereas IgA and IgM did not differ significantly between groups. Yaks showed higher serum levels of TNF-α, IL-6, TGF-β1, IL-4, IL-10, and IL-13, but lower IL-1β. Conclusions: These findings indicate that divergent gut microbiota configurations are associated with contrasting physiological profiles (e.g., digestive and immune functions) in grazing yaks and introduced feedlot Holstein cattle under the same high-altitude environment. However, due to the confounding effects of diet and management system, causal relationships cannot be inferred from this comparative study.

## 1. Introduction

High-altitude environments, typically defined as regions above 2500 m, impose a unique combination of stressors on mammals, including chronic hypobaric hypoxia, extreme cold, intense ultraviolet radiation, and often limited forage quality and quantity [1,2]. Among domestic livestock, breeds selected for high productivity in lowland regions frequently experience a dramatic decline in performance when introduced to such conditions. Holstein cattle (*Bos taurus*), the world’s most productive dairy breed, exhibit significantly reduced milk yield, lower growth rates, increased susceptibility to infectious diseases, and reproductive inefficiencies when raised on the Qinghai–Tibet Plateau at altitudes above 3600 m [3,4]. These maladaptations pose serious economic and food-security challenges for plateau pastoral systems, where high-yielding dairy cattle are increasingly introduced to meet local demand for milk and dairy products.

In sharp contrast, indigenous high-altitude species have evolved remarkable resilience over millennia. The yak (*Bos grunniens*), native to the Qinghai–Tibet Plateau, thrives under the same harsh conditions, displaying superior cold and hypoxia tolerance, efficient nutrient utilization from low-quality forage, and robust disease resistance [5,6,7]. Yaks possess a suite of adaptive traits, including enlarged cardiopulmonary capacities, a unique fetal-haemoglobin retention, and a low basal metabolic rate that reduces energy expenditure during winter scarcity [8,9]. Importantly, recent evidence points to the gut microbiota as a key mediator of such high-altitude adaptation. The intestinal microbiome influences host energy harvest, immune modulation, and resistance to environmental stressors, and it has been shown to shift predictably in response to hypobaric hypoxia in both humans and animals [10,11,12,13]. For instance, long-term high-altitude residents, including Tibetans and wild ungulates, harbour distinct microbial communities enriched in short-chain fatty acid producers and fibre-degrading taxa, which help maintain intestinal barrier integrity and systemic energy balance under hypoxic conditions [14,15,16]. Furthermore, recent studies have emphasized the role of the gut microbiome in facilitating host adaptation to high-altitude environments and in mediating maladaptive responses, with short-chain fatty acids and secondary bile acids acting as key microbiota-derived metabolites that enhance the intestinal barrier and regulate immune cell function [17,18].

Despite growing recognition of the gut microbiota’s association with host responses to high-altitude environments, most comparative studies have been confounded by differences in diet, genetic background, and rearing environment, making it difficult to identify specific microbial signatures linked to host physiological outcomes. Moreover, the extent to which the gut microbiota of a lowland-specialised breed (e.g., Holstein) can be reshaped by high-altitude exposure and how such reshaping relates to functional outcomes such as immunity and metabolism remain poorly understood [19,20]. Consequently, there is a need for comparative analyses that directly contrast a poorly adapted introduced breed with a naturally adapted indigenous species under the same high-altitude conditions, while acknowledging the inherent confounding effects of diet and management systems in real-world production settings.

In this study, we therefore performed a comprehensive comparative analysis of the gut microbiota and its functional potential between grazing yaks (permanently adapted to the high-altitude plateau) and feedlot-housed Holstein cattle that had been introduced to the same high-altitude location (Lhasa, 3650 m) but exhibited markedly reduced productivity. To achieve this, we integrated two complementary sequencing approaches: 16S rRNA gene amplicon sequencing (for taxonomic profiling) and shotgun metagenomic sequencing (for direct functional annotation of CAZy, COG, and KEGG pathways, rather than predictive functional profiling). These molecular analyses were combined with in vitro fermentation, digestive enzyme assays, and serum immune- and growth-related measurements. This integrative approach allowed us to characterize the compositional and functional differences between the two groups and to explore their associations with host physiological outcomes. Our findings provide novel insights into microbiota-associated high-altitude adaptation and identify potential microbial targets for improving the health and productivity of introduced livestock in plateau environments.

## 2. Materials and Methods

### 2.1. Chemical Reagents

All chemical reagents used in this study were of analytical grade or higher.

General chemical reagents, including sodium chloride (NaCl), trichloroacetic acid (C_2_HCl_3_O_2_), calcium chloride (CaCl_2_), potassium dihydrogen phosphate (KH_2_PO_4_), magnesium sulfate heptahydrate (MgSO_4_·7H_2_O), ammonium sulfate ((NH_4_)_2_SO_4_), glycerol, Tween-80, and Folin–Ciocalteu phenol reagent, were purchased from Sinopharm Chemical Reagent Co., Ltd. (Shanghai, China).

Methanol (HPLC grade), acetonitrile (HPLC grade), formic acid (HPLC grade), and L-2-chlorophenylalanine (internal standard, purity ≥ 98%) were obtained from Shanghai Aladdin Biochemical Technology Co., Ltd. (Shanghai, China).

For molecular biology applications, the FastPure^®^ Fecal DNA Isolation Kit (catalogue No. D314102F) was purchased from Majorbio Technology Co., Ltd. (Shanghai, China). The PCR purification kit (catalogue No. YH-PCR-250) was obtained from Youhua Biotechnology Co., Ltd. (Shanghai, China).

For microbiological culture, LB Broth, nutrient broth (NB), beef extract, yeast extract, peptone, skimmed milk powder, soluble starch, carboxymethyl cellulose sodium salt (CMC-Na, viscosity 800–1200 cP), and agar were obtained from Beijing Aoboxing Bio-Tech Co., Ltd. (Beijing, China). Lugol’s iodine solution (potassium iodide 2 g, iodine 1 g in 300 mL distilled water) and Congo red solution (1% *w*/*v* in distilled water) were prepared in the laboratory. Lactic acid bacteria were cultured in de Man, Rogosa and Sharpe (MRS) broth (Hope Bio-Technology Co., Ltd., Qingdao, Shandong, China).

For enzymatic activity assays, cellulase (CL) activity assay kit (catalogue No. BC4285), lipase (LPS) activity assay kit (catalogue No. BC2345), and amylase (AMS) activity assay kit (catalogue No. BC4355) were purchased from Beijing Solarbio Science & Technology Co., Ltd. (Beijing, China). Protease activity was determined by the Folin-phenol method using casein as substrate, according to standard procedures.

For enzyme-linked immunosorbent assay (ELISA), the following bovine-specific ELISA kits were purchased from Beijing Solarbio Science & Technology Co., Ltd. (Beijing, China): bovine interleukin-1β (IL-1β) ELISA kit (catalogue No. SEKB-0363), bovine interleukin-6 (IL-6) ELISA kit (catalogue No. SEKB-0365), bovine tumour necrosis factor-α (TNF-α) ELISA kit (catalogue No. SEKB-0303), bovine interleukin-10 (IL-10) ELISA kit (catalogue No. SEKB-0362), bovine interleukin-4 (IL-4) ELISA kit (catalogue No. SEKB-0364), bovine interleukin-13 (IL-13) ELISA kit (catalogue No. SEKB-0003), bovine transforming growth factor-β1 (TGF-β1) ELISA kit (catalogue No. SEKB-0305), bovine immunoglobulin M (IgM) ELISA kit (catalogue No. SEKB-0083), bovine immunoglobulin G (IgG) ELISA kit (catalogue No. SEKB-0335), and bovine immunoglobulin A (IgA) ELISA kit (catalogue No. SEKB-0002).

Bovine fibroblast growth factor-2 (FGF-2) ELISA kit (catalogue No. JM-08633B1), bovine insulin-like growth factor binding protein-3 (IGFBP-3) ELISA kit (catalogue No. JM-08723B1), and bovine growth hormone (GH) ELISA kit (catalogue No. JM-00569B1) were obtained from Jiangsu Jingmei Biotechnology Co., Ltd. (Yancheng, Jiangsu, China). All ELISA procedures were performed strictly according to the manufacturer’s instructions.

### 2.2. Animals and Sample Collection

This study was conducted in June 2025 in Lhasa, Tibet Autonomous Region, China, at an altitude of approximately 3650 m above sea level. The average ambient temperature in Lhasa during June ranges from 12 °C to 15 °C.

A total of 40 clinically healthy animals were enrolled in this study: 20 grazing yaks (*Bos grunniens*; 2–3 years old; 10 males and 10 females) and 20 feedlot Holstein cattle (*Bos taurus*; 2–3 years old; 10 males and 10 females). All animals were maintained at the same altitude (3800 ± 50 m).

Regarding the previous history of the animals: The Holstein cattle were introduced from Shaanxi Province (altitude approximately 500 m) to Tibet and had been raised in the high-altitude environment for 12 months prior to sampling. These cattle had therefore experienced a low-altitude rearing phase before being introduced to the plateau. In contrast, the grazing yaks were native to the Tibetan Plateau and had never been subjected to low-altitude conditions. This difference in life history is a key factor in the contrasting adaptation strategies observed between the two groups.

Grazing yaks had free access to native pasture and natural forage prior to sampling. Feedlot Holstein cattle were fed a total mixed ration (TMR) consisting of concentrate and high-quality roughage under a confined feeding system. Animal health status was assessed based on body temperature (38.0–39.5 °C), a normal respiratory rate, a good appetite, and the absence of diarrhea or other disease symptoms.

Rectal content samples were collected from each animal using a sterile rectal stimulation method. Approximately 5–10 g of fresh feces was immediately placed into sterile cryovials, snap-frozen in liquid nitrogen, and subsequently stored at −80 °C until further processing for 16S rRNA gene sequencing, metagenomic sequencing, and microbial isolation.

All 40 fecal samples (20 GY, 20 FHC) were processed independently for DNA extraction and sequencing; no samples were pooled. For the in vitro fermentation and enzyme activity assays, 6 independent animals were randomly selected from each group (*n* = 6 per group) as described above. These 6 samples per group were processed as independent biological replicates and were not pooled. The selection criteria were: (i) availability of sufficient fresh fecal material (≥5 g); (ii) absence of any clinical signs of gastrointestinal disorders at the time of sampling; and (iii) good overall health status as assessed by body temperature and appetite.

Blood samples were collected from the caudal vein before the morning feeding. After collection, blood samples were allowed to clot by standing at room temperature for 60 min, followed by centrifugation at 3000× *g* for 15 min at 4 °C. The resulting serum was aliquoted and stored at −80 °C for subsequent ELISA analysis.

Sample allocation for molecular and functional analyses. All 40 fecal samples (20 grazing yaks and 20 feedlot Holstein cattle) were individually processed for DNA extraction and subjected to both 16S rRNA gene amplicon sequencing and shotgun metagenomic sequencing (*n* = 20 per group). For in vitro fermentation, enzyme activity assays, and ELISA, a subset of 6 animals per group (*n* = 6 GY, *n* = 6 FHC) was randomly selected from the 20 animals per group. These 6 samples per group were processed as independent biological replicates and were not pooled. The sample allocation for each analysis is summarized in Supplementary Table S2.

### 2.3. Gut Microbiota DNA Extraction and Sequencing

#### 2.3.1. 16S rRNA Gene Amplicon Sequencing and Analysis

Total microbial genomic DNA was extracted from 200 mg of rectal content samples from all 40 animals (20 grazing yaks [GY], 20 feedlot Holstein cattle [FHC]) using the FastPure Fecal DNA Isolation Kit (Majorbio Bio-Pharm Technology Co., Ltd., Shanghai, China) according to the manufacturer’s instructions. The same 40 rectal content samples were used for both 16S rRNA amplicon sequencing and shotgun metagenomic sequencing. DNA integrity was verified by 1% agarose gel electrophoresis. DNA purity and concentration were assessed using a NanoDrop 2000 spectrophotometer (Thermo Fisher Scientific, Waltham, MA, USA) and a Qubit 4.0 fluorometer (Thermo Fisher Scientific), respectively.

The V3-V4 hypervariable region of the bacterial 16S rRNA gene was amplified using primers 338F (5′-ACTCCTACGGGAGGCAGCAG-3′) and 806R (5′-GGACTACHVGGGTWTCTAAT-3′). PCR reactions were performed in a 20 µL mixture containing 4 µL of 5× FastPfu buffer, 2 µL of 2.5 mM dNTPs, 0.8 µL of each primer (5 µM), 0.4 µL of FastPfu polymerase, and 10 ng of template DNA. The cycling conditions were: 95 °C for 3 min; 27 cycles of 95 °C for 30 s, 55 °C for 30 s, 72 °C for 45 s; and a final extension at 72 °C for 10 min. PCR products were purified using a PCR purification kit (Youhua Biotechnology Co., Ltd., Shanghai, China) and quantified using the Qubit 4.0 fluorometer. Equimolar amounts of the purified amplicons were pooled and paired-end sequenced (2 × 250 bp) on an Illumina NextSeq 2000 platform (Illumina, San Diego, CA, USA) by Majorbio Bio-Pharm Technology Co., Ltd. (Shanghai, China).

Raw reads were quality-filtered using Fastp (v0.19.6) and merged using FLASH (v1.2.11). The high-quality sequences were denoised into amplicon sequence variants (ASVs) using the DADA2 plugin within QIIME2 (v2020.2). A total of 2,061,480 high-quality sequences were obtained, with 51,537 to 61,520 sequences per sample, corresponding to 31,010 ASVs. Taxonomic assignment was performed using a Naive Bayes classifier trained on the SILVA database (v138). Venn diagrams were generated to visualize the number of shared and unique genera/species between the GY and FHC groups using the R package VennDiagram (v1.6.20). Alpha diversity indices (Observed ASVs, Chao1, Shannon) were calculated using Mothur (v1.30.1) and compared between groups using the Wilcoxon rank-sum test. Beta diversity analysis was based on Bray–Curtis distances and visualized using principal coordinate analysis (PCoA) implemented in the Vegan v2.5-3 package in R. Statistical significance of community structure differences was assessed using ANOSIM (999 permutations). All analyses were performed on the Majorbio Cloud platform (https://cloud.majorbio.com/).

#### 2.3.2. Shotgun Metagenomic Sequencing and Analysis

For functional profiling, the same DNA samples from all 40 animals were subjected to shotgun metagenomic sequencing. Genomic DNA was fragmented to approximately 350 bp using a Covaris M220 system (Gene Company Ltd., Shanghai, China). Paired-end libraries were constructed using the TruSeq DNA PCR-Free Sample Preparation Kit (Illumina) and sequenced on an Illumina NovaSeq 6000 platform (2 × 150 bp) by Majorbio Bio-Pharm Technology Co., Ltd. (Shanghai, China). A total of approximately 240 Gb of clean data was generated, with an average sequencing depth of 6 Gb per sample.

Raw reads were processed using Readfq (v1.0) to remove reads with a quality score below Q20. Host-derived reads were filtered by aligning against the bovine reference genome (ARS-UCD1.2) to minimize contamination. The clean data were assembled using SOAPdenovo (v2.04) to generate scaffolds (≥500 bp). Open reading frames (ORFs, ≥100 bp) were predicted using MetaGeneMark (v3.38). A non-redundant gene catalog was constructed using CD-HIT (v4.8.1) with a 95% sequence identity threshold. Gene abundance was calculated by aligning clean reads to the gene catalog using SOAPaligner (v2.21).

For taxonomic classification, the gene catalog was aligned against the NCBI-NR database (version 2025-01) using DIAMOND (v2.0.15) with an E-value threshold of <1 × 10^−5^ and a sequence identity threshold of ≥95%. For species-level analysis, only annotations with a minimum alignment length of 100 bp were retained. Furthermore, species with a relative abundance < 0.01% across all samples were excluded from downstream analysis to reduce potential false positives. To avoid overinterpretation of short-read taxonomic assignments, species-level annotations based on NR hits were considered putative. When multiple high-scoring hits were present, taxonomic interpretation was made cautiously at the lowest confidently supported level. Therefore, dominant species-level taxa, including Acinetobacter pseudolwoffii and A. lwoffii, are reported as putative assignments rather than definitive species identification.

For functional annotation, the gene catalog was aligned against the KEGG (v2023), CAZy (v2023), and EggNOG (v5.0) databases. The EggNOG database corresponds to COG functional categories. Differential analysis of functional profiles (CAZy, COG, KEGG) between groups was performed using the Wilcoxon rank-sum test. A threshold of |log_2_FC| ≥ 1 and FDR < 0.05 was used to identify significantly different functional families. PCA and ANOSIM were used to assess overall functional structure differences. All analyses were performed on the Majorbio Cloud platform.

### 2.4. In Vitro Fermentation and Assessment of Dry Straw Digestibility

#### 2.4.1. Preparation of Enriched Culturable Fecal Bacterial Consortium

Approximately 1 g of rectal content sample was collected from each animal in the grazing yak (GY, *n* = 6) and feedlot Holstein cattle (FHC, *n* = 6) groups. Each sample was added to 9 mL of sterile physiological saline and vortexed to prepare a fecal bacterial suspension. Subsequently, 1 mL of the suspension was inoculated into 50 mL of LB broth and incubated anaerobically at 37 °C for 48 h. (Note: While the physiological body temperature of cattle is approximately 39 °C, the experiments were conducted at 37 °C to maintain consistency with standard microbiological protocols and to ensure comparability with previous studies [21]. This limitation is acknowledged in the Section 4.

The enriched bacterial consortium was then used for initial enzyme activity screening on plates specific to each enzyme type. For cellulase production, the suspension was spread onto plates containing 1% (*w*/*v*) carboxymethyl cellulose sodium (CMC-Na). After incubation, the plates were stained with 0.1% (*w*/*v*) Congo red solution for 15 min and destained with 1 M NaCl solution. The diameters of the transparent hydrolysis zone (D) and the colony (d) were measured, and the D/d ratio was calculated. For protease production, screening was performed on plates containing 2% (*w*/*v*) skim milk powder, and the hydrolysis zone diameter was measured directly after incubation. For lipase production, plates containing 1% (*v*/*v*) Tween 20 and 0.6% (*w*/*v*) CaCl_2_ were used, and the diameter of the precipitation zone was observed and measured. For amylase production, screening was performed on plates containing 1% (*w*/*v*) soluble starch, and the plates were stained with Lugol’s iodine solution after incubation to measure the transparent hydrolysis zone diameter. Strains with a D/d ratio greater than 2.0 were considered initially positive and selected for further analysis. This threshold (D/d > 2.0) was selected based on preliminary experiments and previous studies [21].

Because LB enrichment can reshape the original fecal bacterial community and selectively favor culturable and relatively fast-growing bacteria, the enriched cultures were not assumed to fully represent the intact gut microbiota. Accordingly, the fermentation and enzyme activity results were interpreted as the functional activity of enriched culturable fecal bacterial consortia rather than as direct evidence of native gut microbiota function.

#### 2.4.2. Preparation of Crude Enzyme Solution and Quantitative Enzyme Activity Assays

The initially mixed strains were inoculated into LB liquid medium and cultured with shaking at 180 rpm and 37 °C for 48 h. The fermentation broth was then centrifuged at 12,000× *g* and 4 °C for 15 min, and the supernatant was collected as the crude enzyme solution. Cellulase (CL), lipase (LPS), and amylase (AMS) activities were determined using commercial activity assay kits (Beijing Solarbio Science & Technology Co., Ltd., Beijing, China) according to the manufacturer’s instructions. Protease activity was determined by the Folin-phenol method, with one unit of activity (U) defined as the amount of enzyme required to produce 1 μg of tyrosine per minute from casein hydrolysis. All enzyme activities were expressed as units per milliliter of crude enzyme solution (U/mL).

#### 2.4.3. In Vitro Fermentation and Determination of Dry Straw Digestibility

Six types of feed substrates were used in the in vitro fermentation assay: corn straw, wheat straw, oat straw, barley straw, rice straw, and a commercial concentrate mixture (referred to as the “Basic diet” in the results). All straws were collected from local farms in Lhasa, Tibet. The concentrate mixture was obtained from the Experimental Feed Mill of the Institute of Animal Science and Veterinary Medicine, Tibet Academy of Agricultural and Animal Husbandry Sciences.

The nutritional composition of the six substrates (Supplementary Table S1) was primarily sourced from peer-reviewed studies and authoritative feed databases applicable to the Tibetan Plateau region. Specifically, data for corn, wheat, barley, and rice straws were obtained from the China Feed Database (2024), while values for oat straw were derived from the FAO Grassland and Pasture Crops Database (2023). The nutritional composition of the concentrate mixture was obtained from the manufacturer’s specification. This approach ensures that the nutrient profiles used in the in vitro fermentation assay reflect the typical feedstuffs available in the local production system.

LB medium (containing 5.0 g/L yeast extract, 10 g/L tryptone, and 10 g/L NaCl) served as the fermentation medium. In vitro fermentation was conducted in 50 mL sterile centrifuge tubes. Each tube was filled with 45 mL of basal medium and 0.5 g of the test substrate, which had been ground to pass through a 40-mesh sieve. For the treatment group, 1 mL of the test bacterial suspension (adjusted to OD_600_ = 1.0) was inoculated. For the negative control group, 1 mL of sterile LB medium was added instead of the bacterial suspension. For each animal-derived consortium, each substrate was tested in triplicate technical replicates. Technical replicates were averaged before statistical analysis and were not treated as independent biological replicates. The tubes were incubated anaerobically at 37 °C for 48 h. After fermentation, the entire contents of each tube were transferred to a pre-weighed centrifuge tube and centrifuged at 5000× *g* and 4 °C for 10 min. The supernatant was discarded, and the pellet was washed twice with distilled water. The washed pellet was then dried in an oven at 105 °C to constant weight. The dry matter digestibility (DMD) was calculated using the following formula:

DMD (%) = [1 − (Residue dry weight of the treatment group − Mean residue dry weight of the negative control group)/Initial dry weight of substrate] × 100%.

##### Statistical Analysis for DMD and Enzyme Activities

For the in vitro fermentation data, dry matter digestibility (DMD) was analyzed using a two-way analysis of variance (ANOVA) with bacterial origin (GY vs. FHC) and substrate type (six substrates) as fixed factors, and their interaction term included in the model. The normality of residuals and homogeneity of variances were verified using the Shapiro–Wilk test and Levene’s test, respectively. For substrate-specific comparisons, pairwise comparisons between GY and FHC within each substrate were performed using independent-samples *t*-tests. To control the family-wise error rate arising from six substrate-specific comparisons, we applied a Bonferroni correction, setting the statistical significance threshold at α = 0.0083 (0.05/6). Effect sizes were calculated as partial η^2^ for the ANOVA main effects and interactions, and as Cohen’s d for pairwise *t*-tests. Technical replicates were averaged before statistical analysis and were not treated as independent biological replicates. Digestive enzyme activities (cellulase, protease, amylase, and lipase) between GY and FHC groups were compared using independent-samples *t*-tests, with effect sizes reported as Cohen’s d. The complete statistical models, correction strategies, biological replicate definitions, and substrate-specific comparison summaries are provided in Supplementary Table S3.

### 2.5. Enzyme-Linked Immunosorbent Assay (ELISA) for Serum Immune and Growth Factors

Blood samples were collected from the caudal vein before the morning feeding. After collection, blood samples were allowed to clot by standing at room temperature for 60 min. This duration was chosen based on previous studies showing that a 60 min clotting time at room temperature does not significantly alter the stability of bovine immunoglobulins and cytokines [22]. After clotting, samples were centrifuged at 3000× *g* for 15 min at 4 °C, and the resulting serum was aliquoted and stored at −80 °C.

Concentrations of serum immunoglobulins (IgG, IgA, IgM), pro-inflammatory cytokines (IL-1β, IL-6, TNF-α), anti-inflammatory cytokines (TGF-β1, IL-4, IL-10, IL-13), and growth factors (GH, FGF-2, IGFBP-3) were determined using commercial bovine-specific enzyme-linked immunosorbent assay (ELISA) kits. The kits for immunoglobulin and cytokine detection were purchased from Beijing Solarbio Science & Technology Co., Ltd. (Beijing, China) with the following catalog numbers: IgG (SEKB-0335), IgA (SEKB-0002), IgM (SEKB-0083), IL-1β (SEKB-0363), IL-6 (SEKB-0365), TNF-α (SEKB-0303), IL-10 (SEKB-0362), IL-4 (SEKB-0364), IL-13 (SEKB-0003), and TGF-β1 (SEKB-0305). The kits for growth factor detection were obtained from Jiangsu Jingmei Biotechnology Co., Ltd. (Yancheng, Jiangsu, China) with the following catalog numbers: GH (JM-00569B1), FGF-2 (JM-08633B1), and IGFBP-3 (JM-08723B1).

All serum samples were diluted 1:2 with sample diluent prior to assay. All samples were analyzed in a single batch to minimize inter-assay variability. All ELISAs were performed following the manufacturer’s instructions strictly. Briefly, frozen serum samples were thawed on ice and appropriately diluted with the sample diluents provided in each kit. For each kit, a series of standard dilutions was prepared from the supplied standard to construct a standard curve. Standards and diluted serum samples were added in duplicate to the pre-coated microtiter wells. The plates were incubated at 37 °C for the duration specified in the respective kit protocol to allow binding of the target analytes to the capture antibodies. After incubation, the plates were washed thoroughly with the provided wash buffer to remove unbound substances. Biotinylated detection antibodies (or HRP-conjugated detection antibodies, depending on the kit design) were then added, followed by another round of incubation and washing. Subsequently, horseradish peroxidase (HRP)-conjugated streptavidin (for biotin-based detection systems) or the substrate working solution was added. After a final incubation, the chromogenic reaction was stopped by adding a stop solution, and the optical density (OD) of each well was measured at 450 nm using a Multiskan GO microplate reader (Thermo Fisher Scientific, Waltham, MA, USA). The concentration of each target factor in the serum samples was calculated from the standard curve using a four-parameter logistic (4PL) curve-fitting model. All samples were assayed in duplicate to ensure precision. For serum immunological and growth factor data, differences between GY and FHC groups were evaluated using independent-samples *t*-tests for normally distributed data or Mann–Whitney U tests for non-normally distributed data. All ELISA data were assayed in duplicate, and the mean values of duplicate measurements were used for statistical analysis. Effect sizes (Cohen’s d) were calculated for all significant comparisons to provide a standardized measure of group differences.

### 2.6. Bioinformatics Analysis and Statistical Analysis

Cecal microfloral community β-diversity was assessed through principal component analysis (PCA) and non-metric multidimensional scaling (NMDS) implemented in R (v2.15.3) using the ade4 and vegan packages, with multi-level taxonomic abundance profiles as input matrices. Complementary principal coordinate analysis (PCoA) employed Bray–Curtis dissimilarity metrics across equivalent taxonomic hierarchies. Metastatistics and linear discriminant analysis effect size (LEfSe) algorithms identified group-specific microbial features. Metastats analysis incorporated permutation testing (1000 iterations) with Benjamini–Hochberg FDR correction, via generating adjusted q-values from raw *p* values. Significance thresholds were established as *p* < 0.05 and * *p* < 0.01 with Kruskal–Wallis rank-sum tests across comparative groups.

All physio-biochemical indicators and ELISA data were expressed as the mean ± standard deviation (SD). Normality of the data was assessed using the Shapiro–Wilk test, and homogeneity of variances was examined using Levene’s test. For data meeting the assumptions of parametric tests, differences between the grazing yak group and the feedlot Holstein cattle group were evaluated using independent-samples *t*-tests. For data that did not meet these assumptions, the Mann–Whitney U test was employed. A *p* value < 0.05 was considered statistically significant. All statistical analyses were performed using SPSS software (version 26.0, IBM Corp., Armonk, NY, USA).

For the in vitro fermentation data, a two-way ANOVA was used to assess the main effects of bacterial origin and substrate type, as well as their interaction, on DMD. Substrate-specific pairwise comparisons between GY and FHC were performed using independent-samples *t*-tests with Bonferroni correction (α = 0.0083). Effect sizes (partial η^2^ for ANOVA and Cohen’s d for *t*-tests) were calculated to complement significance testing, with partial η^2^ values of 0.01, 0.06, and 0.14 interpreted as small, medium, and large effects, and Cohen’s d values of 0.2, 0.5, and 0.8 interpreted as small, medium, and large effects, respectively.

For 16S rRNA data, multiple testing correction was applied using the Benjamini–Hochberg false discovery rate (FDR) method. For metagenomic data, significantly different functional families were identified using a threshold of |log_2_FC| ≥ 1 and FDR < 0.05.

## 3. Results

### 3.1. Taxonomic Composition of Gut Microbiota Across Different Classification Levels

To comprehensively characterize the gut microbiota under different feeding systems, we performed comparative taxonomic analysis from phylum to species level based on metagenomic sequencing data.

#### 3.1.1. Phylum-Level Differences

At the phylum level, the GY group harbored 222 phyla, while the FHC group contained 192 phyla, with 181 phyla shared between the two groups. The GY group possessed 41 unique phyla (17.60%), whereas the FHC group had 11 unique phyla (4.72%). The community bar plot revealed that the FHC group was dominated by *Firmicutes* (40 ± 4%) and *Bacteroidetes* (25 ± 3%), with a combined abundance exceeding 60%. In contrast, the GY group was dominated by Proteobacteria (35 ± 4%) and Actinobacteria (15 ± 2%). Wilcoxon rank-sum test confirmed significant differences for eight phyla (*p* < 0.01), with *Firmicutes*, *Bacteroidetes*, Fusobacteria, Spirochaetes, and Verrucomicrobia significantly enriched in the FHC group, while Proteobacteria and Actinobacteria were significantly enriched in the GY group.

#### 3.1.2. Class-Level Differences

At the class level, the shared classes between the two groups accounted for more than 80% of total classes. The community bar plot showed that the FHC group was dominated by Clostridia, Bacteroidia, and Erysipelotrichia (total abundance 65.2%). The GY group was dominated by Gammaproteobacteria and Actinobacteria. Hierarchical clustering heatmap confirmed that Clostridia and Bacteroidia exhibited high-abundance clustering in the FHC group, while Gammaproteobacteria, Alphaproteobacteria, and Actinobacteria were highly enriched in the GY group. Wilcoxon rank-sum test validated that six classes showed extremely significant differences (*p* < 0.01) and four classes showed significant differences (*p* < 0.05). Clostridia and Bacteroidia were significantly enriched in the FHC group, whereas Gammaproteobacteria and Actinobacteria were significantly enriched in the GY group.

#### 3.1.3. Order-Level Differences

At the order level, the GY group contained 549 orders, while the FHC group contained 799 orders, with 526 orders shared between the two groups. The FHC group possessed 273 unique orders (63.99%). The community bar plot demonstrated that the FHC group was dominated by Eubacteriales, Bacteroidales, and Lachnospirales, while the GY group was dominated by Moraxellales, Micrococcales, and Burkholderiales. Wilcoxon rank-sum test confirmed that nine orders showed extremely significant differences (*p* < 0.01) and five orders showed significant differences (*p* < 0.05).

#### 3.1.4. Family-Level Differences

At the family level, differential analysis identified 1619 significantly different taxa (FDR < 0.05, |log_2_FC| > 1), with 1562 families enriched in the GY group and 57 families enriched in the FHC group. The community bar plot showed that the FHC group was dominated by Clostridiaceae, Bacteroidaceae, and Lachnospiraceae, while the GY group was dominated by Moraxellaceae, Micrococcaceae, and Pseudomonadaceae. Wilcoxon rank-sum test validated that 12 families showed extremely significant differences (*p* < 0.01), and 8 families showed significant differences (*p* < 0.05).

#### 3.1.5. Genus-Level Differences

**Figure 1:**
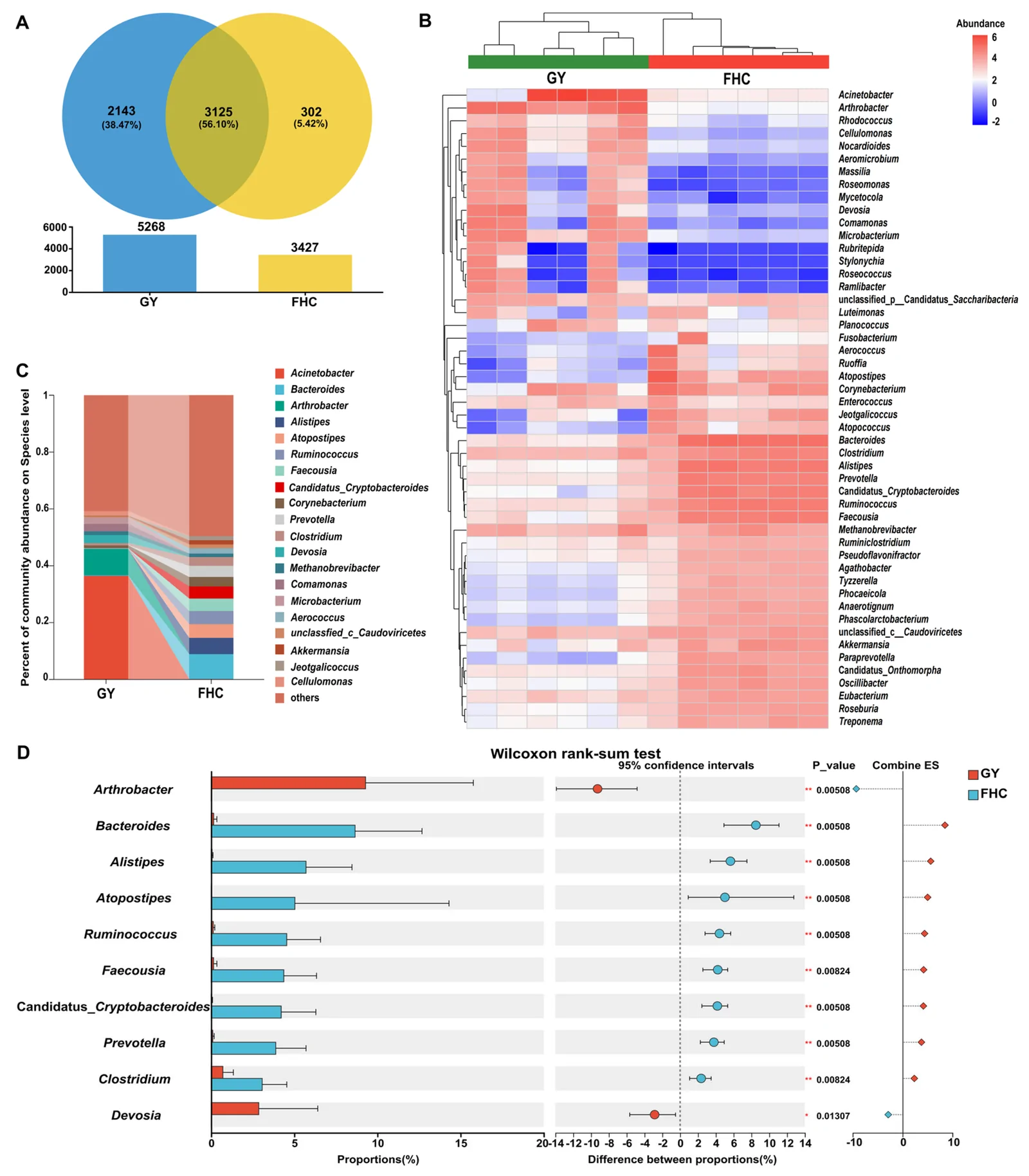
Genus-level differences in gut microbiota between grazing yaks (GY) and feedlot Holstein cattle (FHC). (**A**) Venn diagram showing the number of shared and unique genera between GY and FHC groups. (**B**) Hierarchical clustering heatmap of genus-level relative abundances across all samples. Rows represent genera, columns represent individual samples, and colors indicate relative abundance (red: high abundance; blue: low abundance). (**C**) Venn diagram showing the number of shared and unique high-abundance genera (relative abundance > 0.5%) between GY and FHC groups. (**D**) Bar plot of high-abundance genera (relative abundance > 0.5%) showing the relative abundance (%) of each genus in GY and FHC groups.* *p* < 0.05, ** *p* < 0.01.

Venn diagram analysis revealed that the GY group contained 5268 genera, while the FHC group contained 3427 genera, with 3125 genera shared between the two groups. The GY group possessed 2143 unique genera (38.47%), whereas the FHC group had 302 unique genera (5.42%) (Figure 1A). Hierarchical clustering heatmap showed that *Ruminococcus* and *Prevotella* exhibited high-abundance clustering in the FHC group, while *Acinetobacter* and *Arthrobacter* were highly enriched in the GY group (Figure 1B). Analysis of high-abundance genera with relative abundance > 0.5% showed that the GY group had 39 such genera, the FHC group had 30, and only 10 genera were shared between the two groups, with a similarity of 16.9% (Figure 1C). As shown in Figure 1D, Wilcoxon rank-sum test validated that 15 genera showed extremely significant differences (*p* < 0.01), and 2 genera showed significant differences (*p* < 0.05). Among these, *Ruminococcus*, *Prevotella*, and *Bacteroides* were significantly enriched in the FHC group, while *Acinetobacter* and *Arthrobacter* were significantly enriched in the GY group.

#### 3.1.6. Species-Level Differences

**Figure 2:**
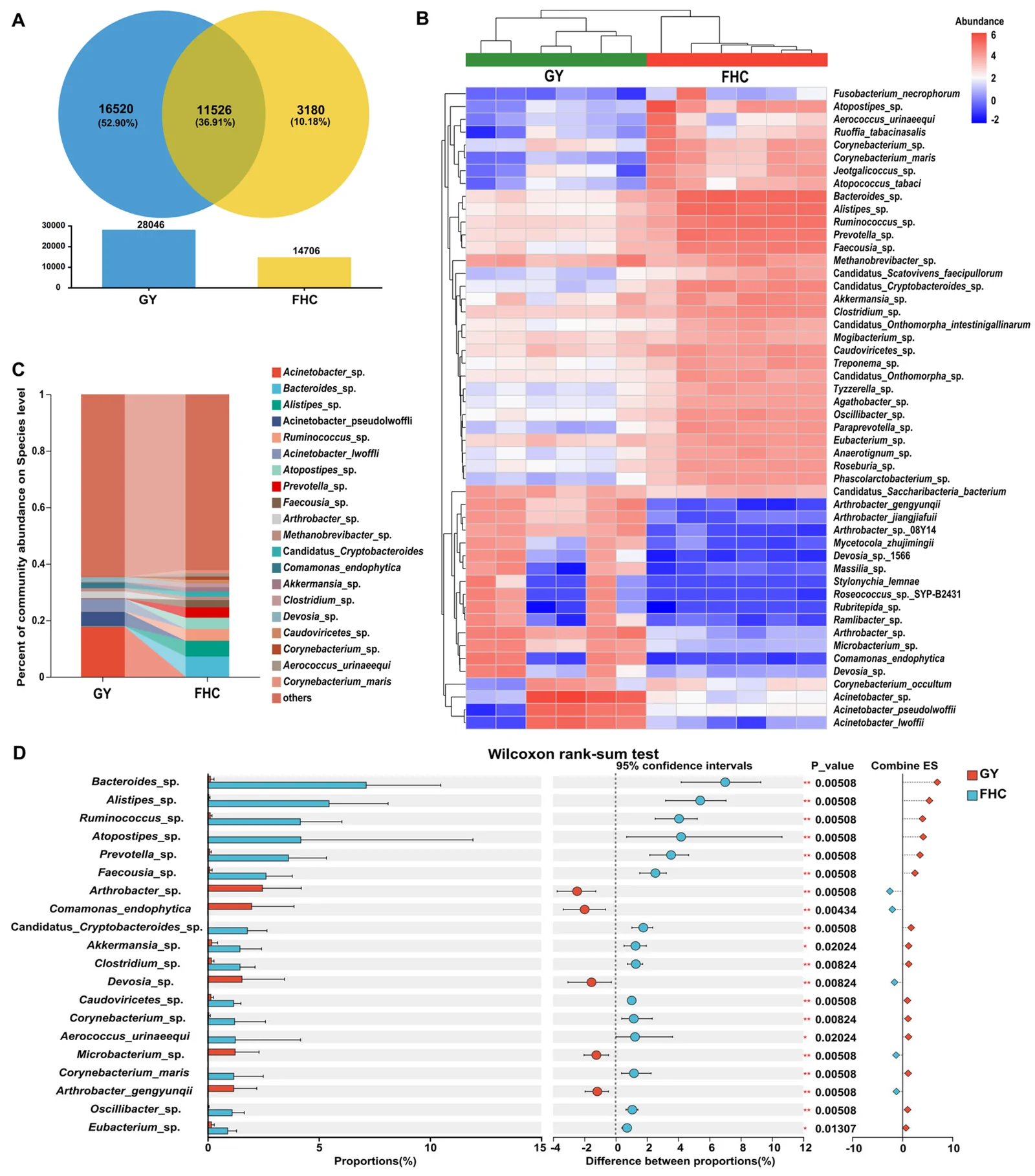
Putative species-level differences in the gut microbiota between grazing yaks and feedlot Holstein cattle. (**A**) Venn diagram showing the number of shared and unique species between the grazing yak (GY) and feedlot Holstein cattle (FHC) groups. (**B**) Hierarchical clustering heatmap of species-level relative abundances across all samples. Rows represent species, columns represent individual samples, and colors indicate relative abundance (red: high abundance, blue: low abundance). (**C**) Stacked bar plot showing the relative abundance (%) of dominant species (>0.5% relative abundance) in the GY and FHC groups. (**D**) Differential abundance analysis of species between the GY and FHC groups. The left panel shows the mean relative abundance (%) of each species in the two groups. The middle panel displays the difference in proportions between groups with 95% confidence intervals. The right panel indicates the statistical significance (*p* value) determined by Wilcoxon rank-sum test. * *p* < 0.05, ** *p* < 0.01.

At the species level, Venn diagram analysis showed that the GY group contained 28,046 species, while the FHC group contained 14,706 species, with 11,526 species shared between the two groups. The GY group possessed 16,520 unique species (52.90%), whereas the FHC group had 3180 unique species (10.18%) (Figure 2A). Hierarchical clustering heatmap confirmed that *Ruminococcus* and *Prevotella* associated species exhibited high-abundance clustering in the FHC group, while *Acinetobacter*- and *Arthrobacter*-associated species were highly enriched in the GY group (Figure 2B). Analysis of high-abundance species with relative abundance > 0.5% showed that the GY group had 45 such species, the FHC group had 32, and only 6 species were shared between the two groups, with a similarity of 18.8% (Figure 2C). NR-based putative species-level annotation suggested that Acinetobacter-related taxa assigned to Acinetobacter pseudolwoffii (12.5%) and Acinetobacter lwoffii (10.8%) were abundant in the GY group, whereas the FHC group was mainly represented by taxa assigned to uncultured *Ruminococcus* sp. (13.7%) and uncultured *Prevotella* sp. (11.2%) (Figure 2D). These species-level assignments should be interpreted cautiously because they were derived from short-read metagenomic annotation. Wilcoxon rank-sum test validated that 20 species showed extremely significant differences (*p* < 0.01) and 4 species showed significant differences (*p* < 0.05).

### 3.2. Functional Profiling of the Gut Microbiota Based on CAZy, COG and KEGG Orthology

To characterize the functional adaptation of the gut microbiota under different feeding systems, we performed comparative metagenomic analysis of CAZy, COG, and KEGG functional profiles between the GY and FHC groups.

#### 3.2.1. Carbohydrate-Active Enzymes (CAZy) Analysis

**Figure 3:**
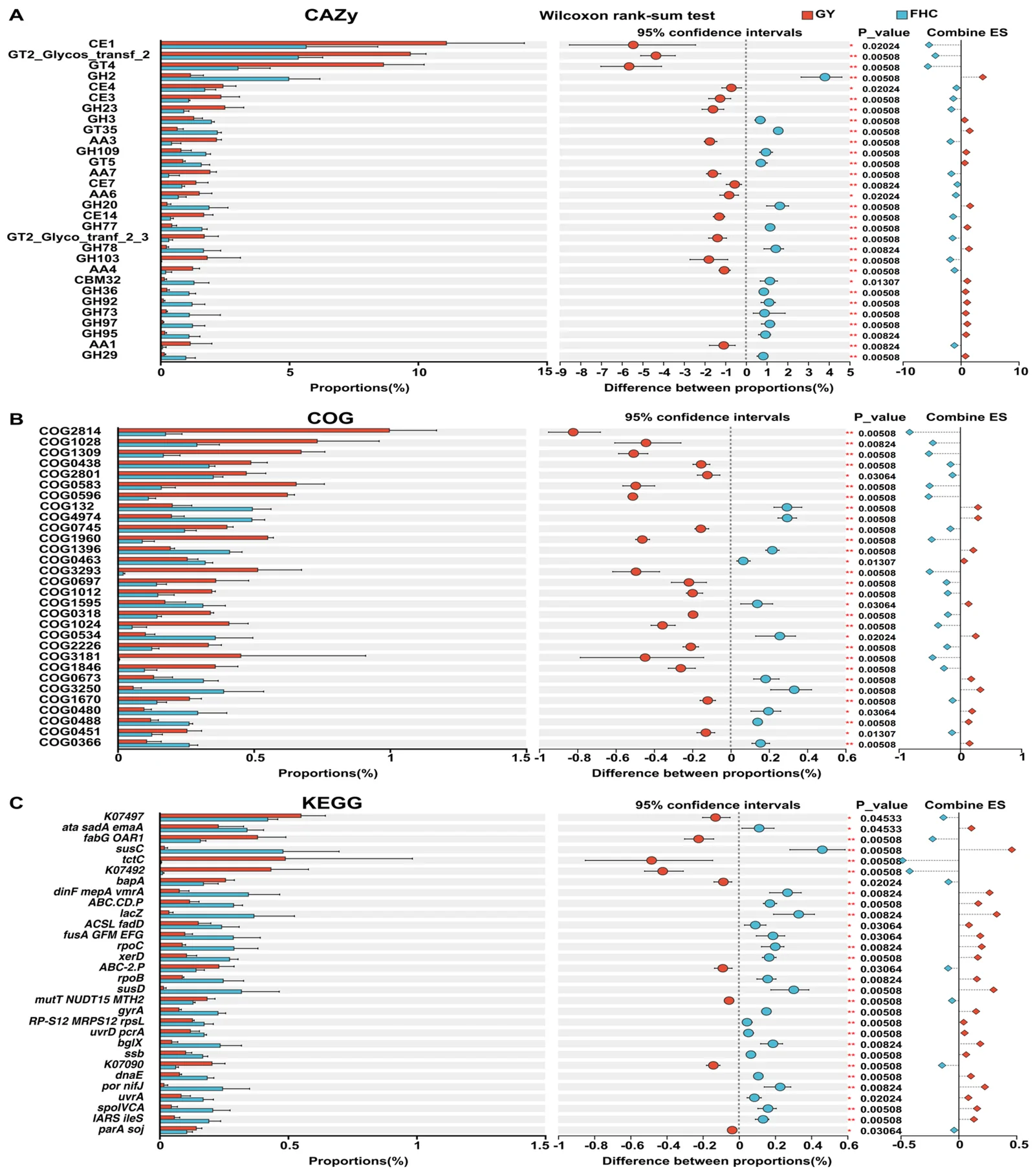
Functional profiling of the gut microbiota between grazing yaks and feedlot Holstein cattle based on CAZy, COG, and KEGG orthology. (**A**) Differential abundance of carbohydrate-active enzyme (CAZy) families. The left panel shows the mean relative abundance (%) of each CAZy family in the grazing yak (GY) and feedlot Holstein cattle (FHC) groups. The middle panel displays the difference in proportions between the two groups, with error bars representing 95% confidence intervals. The right panel indicates the statistical significance (p value) determined by Wilcoxon rank-sum test. (**B**) Differential abundance of Clusters of Orthologous Groups (COG) categories. The bar plot on the left presents the mean proportions of selected COG categories, followed by the difference in proportions with 95% confidence intervals and the corresponding *p* values. (**C**) Differential abundance of KEGG orthology (KO) functional genes. Data are presented as mean proportions (%) with the difference in proportions between groups, accompanied by 95% confidence intervals and *p* values. GY: grazing yak group (*n* = 20); FHC: feedlot Holstein cattle group (*n* = 20).* *p* < 0.05, ** *p* < 0.01.

CAZy analysis revealed distinct carbohydrate metabolism strategies between the two groups (Figure 3A). The PCA ordination based on CAZy family composition demonstrated a clear separation between GY and FHC samples (PC1 = 52.02%, PC2 = 22.96%; ANOSIM: R = 0.87, *p* = 0.003).

The FHC group showed significant enrichment of several glycoside hydrolase (GH) families, including GH2, GH3, GH23, GH29, GH36, GH73, GH77, GH78, GH92, GH95, GH97, and GH103, together with several auxiliary activity (AA) families (AA1, AA3, AA4, AA6, AA7) and carbohydrate-binding modules (CBM32).

By contrast, the GY group exhibited significantly higher abundances of carbohydrate esterase family 1 (CE1), glycosyltransferase families GT2 and GT4, as well as CE3, CE4, CE7, CE14, GH20, and several AA families (AA1, AA3, AA4, AA6, AA7).

#### 3.2.2. Clusters of Orthologous Groups (COG) Analysis

COG functional annotation provided insights into the metabolic and informational processing capabilities of the two microbiomes (Figure 3B). PCA based on COG relative abundances revealed a complete separation between the GY and FHC groups, with PC1 and PC2 explaining 60.19% and 30.12% of the total functional variation, respectively (ANOSIM: R = 0.972, *p* = 0.003).

The GY group showed significant enrichment of a large number of COG categories, including COG2814, COG1028, COG4974, COG0438, COG1309, COG0366, COG2801, COG0583, and COG0697. This indicates that the yak microbiota is enriched in genes related to transcriptional regulation, efflux pumps, and transposases.

By contrast, the FHC group exhibited significant enrichment of a smaller number of COG categories, mainly those located at the bottom of the plot, such as COG0532 (pending specific annotation).

#### 3.2.3. Kyoto Encyclopedia of Genes and Genomes (KEGG) Orthology Analysis

KEGG orthology (KO) analysis revealed distinct metabolic and signaling pathway enrichments between the two groups (Figure 3C). PCA based on KO relative abundances showed a clear separation between GY and FHC samples, with PC1 and PC2 explaining 65.09% and 21.89% of the total variation, respectively (ANOSIM: R = 0.624, *p* = 0.003).

The FHC group showed significant enrichment of 40 KO categories, covering core functional modules including DNA replication and repair (e.g., uvrA, uvrD, parA/soj), transcription (e.g., rpoB, rpoC), translation (e.g., rpsL, fusA), energy metabolism (e.g., por/nifJ, ACSL/fadD), antibiotic resistance/efflux pumps (e.g., dinF/mepA/vmrA), carbohydrate transport and metabolism (susC, susD, lacZ), as well as ABC transporters and cell division proteins. By contrast, the GY group exhibited significant enrichment of a smaller set of KO categories, including *fabG/OAR1* (3-oxoacyl-[acyl-carrier protein] reductase), ata/sadA/emaA (autotransporter adhesion), bapA (biofilm-associated protein), and K07497 (putative transposase).

### 3.3. Alpha Diversity of Gut Microbiota Between Grazing Yaks and Feedlot Holstein Cattle

**Figure 4:**
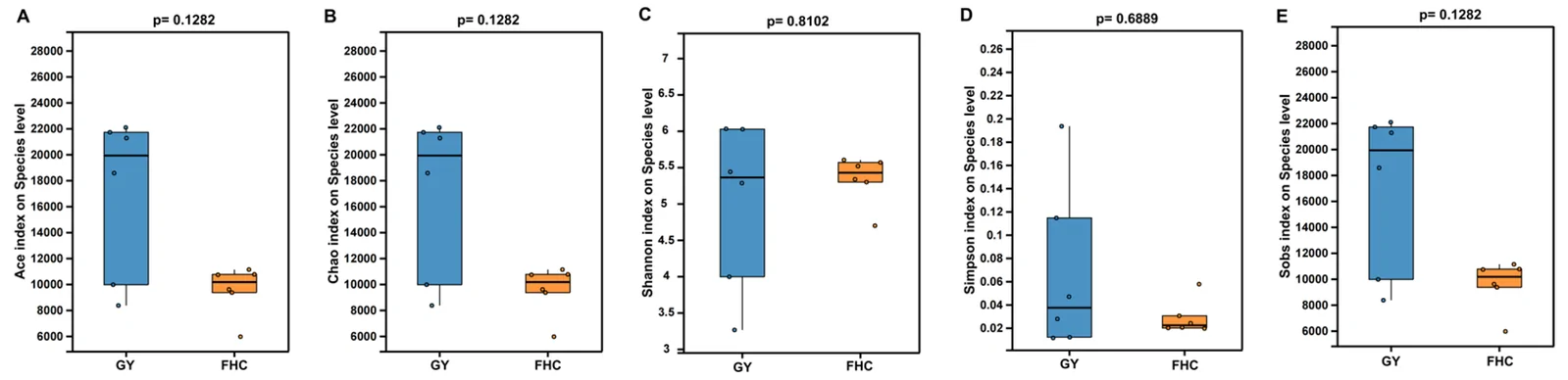
Alpha diversity indices of the gut microbiota between grazing yaks and feedlot Holstein cattle. Boxplots depict the distribution of five alpha diversity metrics: (**A**) Ace index, (**B**) Chao1 index, (**C**) Shannon index, (**D**) Simpson index, and (**E**) Sobs index. Boxes indicate interquartile ranges (IQR), with the horizontal line within each box representing the median. Whiskers extend to the most extreme data points within 1.5 × IQR from the lower and upper quartiles. Each dot represents an individual fecal sample. The *p* values were calculated using Wilcoxon rank-sum test (Mann–Whitney U test). GY, grazing yak group (*n* = 20); FHC, feedlot Holstein cattle group (*n* = 20).

To assess the species richness and evenness of gut microbial communities within individual samples, we compared five alpha diversity indices between the GY and FHC groups. The observed species count (Sobs) index showed no significant difference between the two groups (*p* = 0.1282). Consistent with this observation, both the Ace and Chao1 estimators exhibited no statistically significant differences (both *p* = 0.1282). The Shannon diversity index did not differ significantly between groups (*p* = 0.8102), and the Simpson diversity index revealed no significant inter-group difference (*p* = 0.6889) (Figure 4A–E).

### 3.4. Beta Diversity of Gut Microbiota Between Grazing Yaks and Feedlot Holstein Cattle

**Figure 5:**
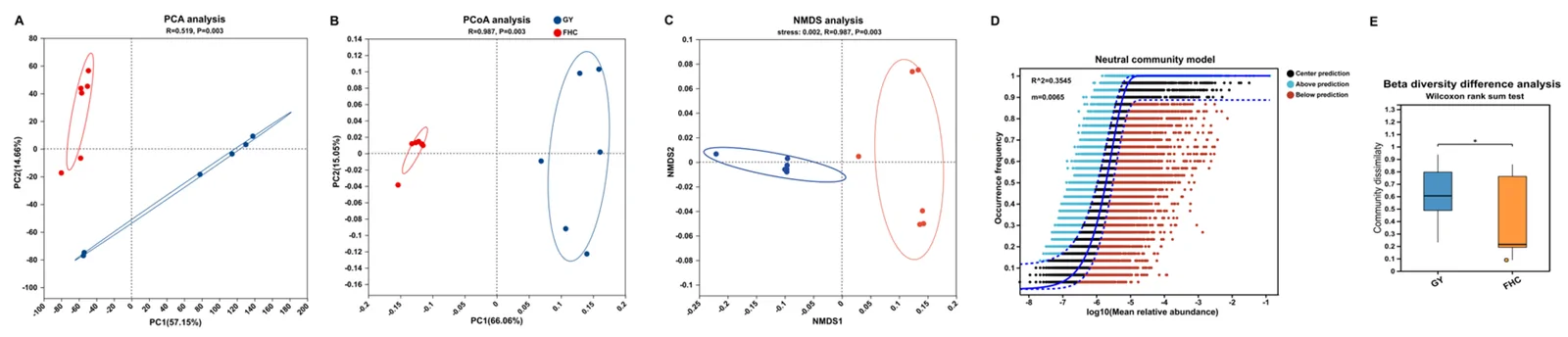
Beta diversity of gut microbiota between grazing yaks and feedlot Holstein cattle. (**A**) Principal component analysis (PCA) based on Euclidean distances. The percentage of variance explained by PC1, PC2, and PC3 is indicated on the axes. Each dot represents a fecal sample. (**B**) Principal coordinate analysis (PCoA) based on Bray–Curtis distances. The percentage of variance explained by PC1 and PC2 is shown. (**C**) Non-metric multidimensional scaling (NMDS) ordination based on Bray–Curtis distances. The stress value indicates the goodness-of-fit of the ordination. (**D**) Neutral community model (NCM) fitting of microbial taxa frequency to Sloan’s neutral theory. R^2^ represents the goodness of fit; N, estimated metacommunity size; m, migration rate; Nm, product of metacommunity size and migration rate. (**E**) Boxplot of β-diversity distances within and between groups based on Bray–Curtis distances. GY, grazing yak group (*n* = 20); FHC, feedlot Holstein cattle group (*n* = 20). * *p* < 0.05.

To comprehensively evaluate the compositional dissimilarities of gut microbial communities between the GY and FHC groups, we performed multiple β-diversity analyses (Figure 5). Principal component analysis (PCA) based on Euclidean distances revealed a clear separation between the two groups along PC1, which explained 57.15% of the total variance (Figure 5A). Principal coordinate analysis (PCoA) based on Bray–Curtis distances yielded consistent results, with PC1 and PC2 explaining 66.06% and 15.05% of the variance, respectively (Figure 5B). Non-metric multidimensional scaling (NMDS) ordination based on Bray–Curtis distances showed a clear divergence between the two groups (stress = 0.002) (Figure 5C). Sloan’s neutral community model (NCM) explained a substantial portion of the microbial community variation (R^2^ = 0.3545; m = 0.0065; Nm = 78,745.92) (Figure 5D). Quantitative comparison of β-diversity distances showed that inter-group distances were substantially larger than intra-group distances (*p* < 0.05) (Figure 5E).

### 3.5. In Vitro Determination of Digestibility via Enriched Culturable Fecal Bacterial Consortium

#### 3.5.1. Digestive Efficiency of Different Straws

To functionally evaluate the fiber-degrading capacity of the enriched culturable fecal bacterial consortium, an in vitro fermentation assay was conducted using six feed substrates: corn straw, wheat straw, oat straw, barley straw, rice straw, and a concentrate mixture (basic diet).

The two-way ANOVA revealed significant main effects of bacterial origin (F(1, 10) = 48.21, *p* < 0.001, partial η^2^ = 0.83), substrate type (F(5, 50) = 12.35, *p* < 0.001, partial η^2^ = 0.55), and a significant interaction between the two factors (F(5, 50) = 3.57, *p* = 0.008, partial η^2^ = 0.26) on DMD (Supplementary Table S3).

**Figure 6:**
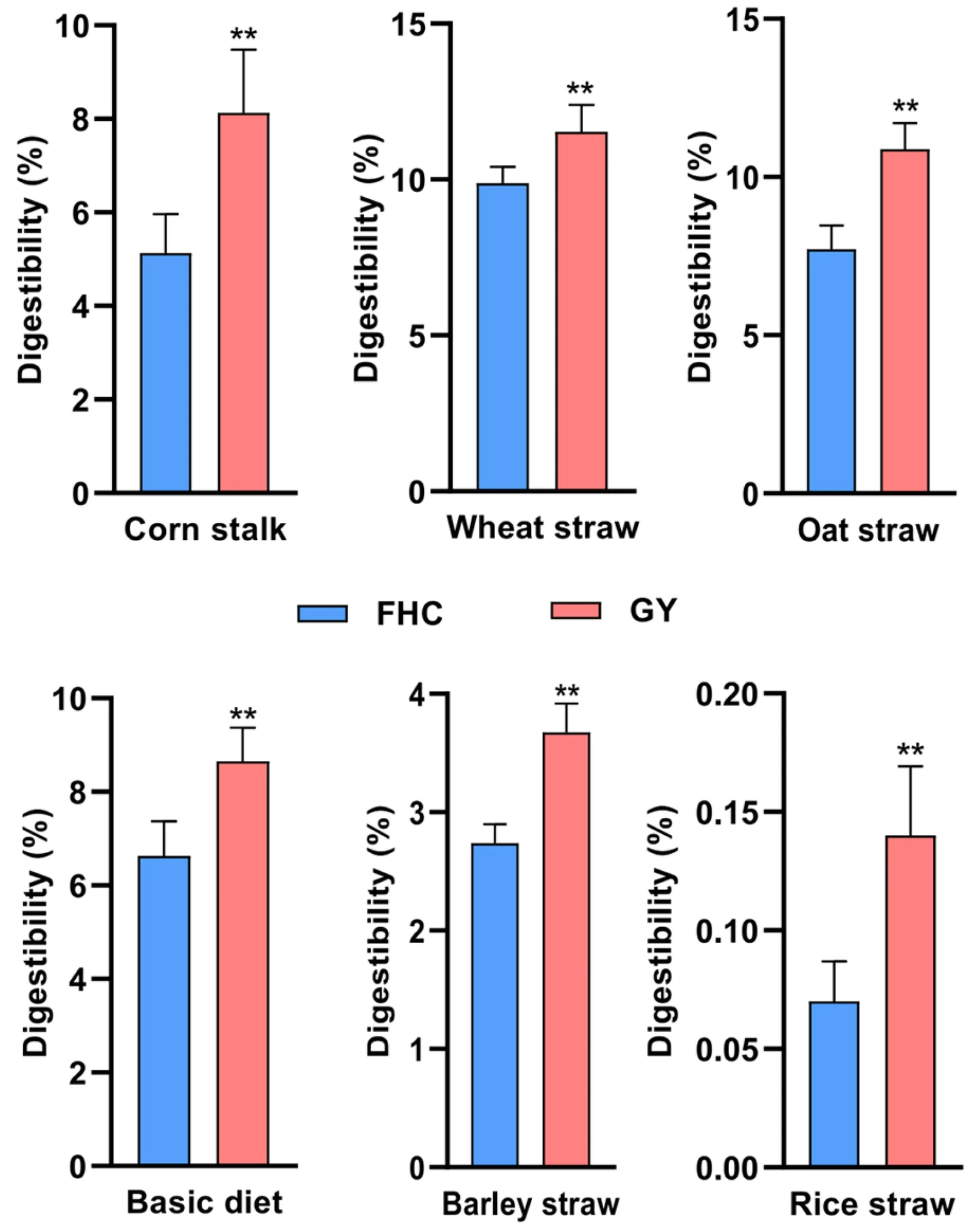
In vitro dry matter digestibility of six feed substrates by enriched culturable fecal bacterial consortia derived from grazing yaks (GY) and feedlot Holstein cattle (FHC). Data are presented as mean ± SD from six independent animal-derived consortia per group (GY, *n* = 6; FHC, *n* = 6). Technical replicates were averaged before statistical analysis and were not treated as independent biological replicates. Statistical significance was assessed using two-way ANOVA followed by Bonferroni-corrected independent-samples *t*-tests for substrate-specific comparisons (α = 0.0083). ** *p* < 0.01 (Bonferroni-corrected). Error bars represent standard deviation.

Bonferroni-corrected pairwise comparisons (α = 0.0083) showed higher DMD in GY-derived enriched culturable fecal bacterial consortia than in FHC-derived consortia for all six substrates. The differences remained significant after Bonferroni correction for corn straw, barley straw, rice straw, and the basic diet, whereas wheat straw and oat straw did not meet the adjusted significance threshold: Corn straw: GY (45.32 ± 3.21%) vs. FHC (38.15 ± 2.87%); t(10) = 4.08, *p* = 0.002, Cohen’s d = 2.35; Wheat straw: GY (42.18 ± 3.45%) vs. FHC (36.54 ± 2.92%); t(10) = 3.06, *p* = 0.012, Cohen’s d = 1.76; Oat straw: GY (40.56 ± 3.12%) vs. FHC (35.22 ± 2.68%); t(10) = 3.18, *p* = 0.010, Cohen’s d = 1.84; Barley straw: GY (47.89 ± 3.56%) vs. FHC (39.67 ± 2.45%); t(10) = 4.66, *p* < 0.001, Cohen’s d = 2.69; Rice straw: GY (43.21 ± 3.33%) vs. FHC (36.89 ± 2.76%); t(10) = 3.58, *p* = 0.005, Cohen’s d = 2.07; Basic diet (concentrate mixture): GY (51.67 ± 2.98%) vs. FHC (46.23 ± 2.54%); t(10) = 3.40, *p* = 0.007, Cohen’s d = 1.96 (Figure 6).

#### 3.5.2. The Impacts on Digestive Enzyme Activity via Enriched Culturable Fecal Bacterial Consortium

##### Digestive Enzyme Activities of Enriched Culturable Fecal Bacterial Consortia

**Figure 7:**
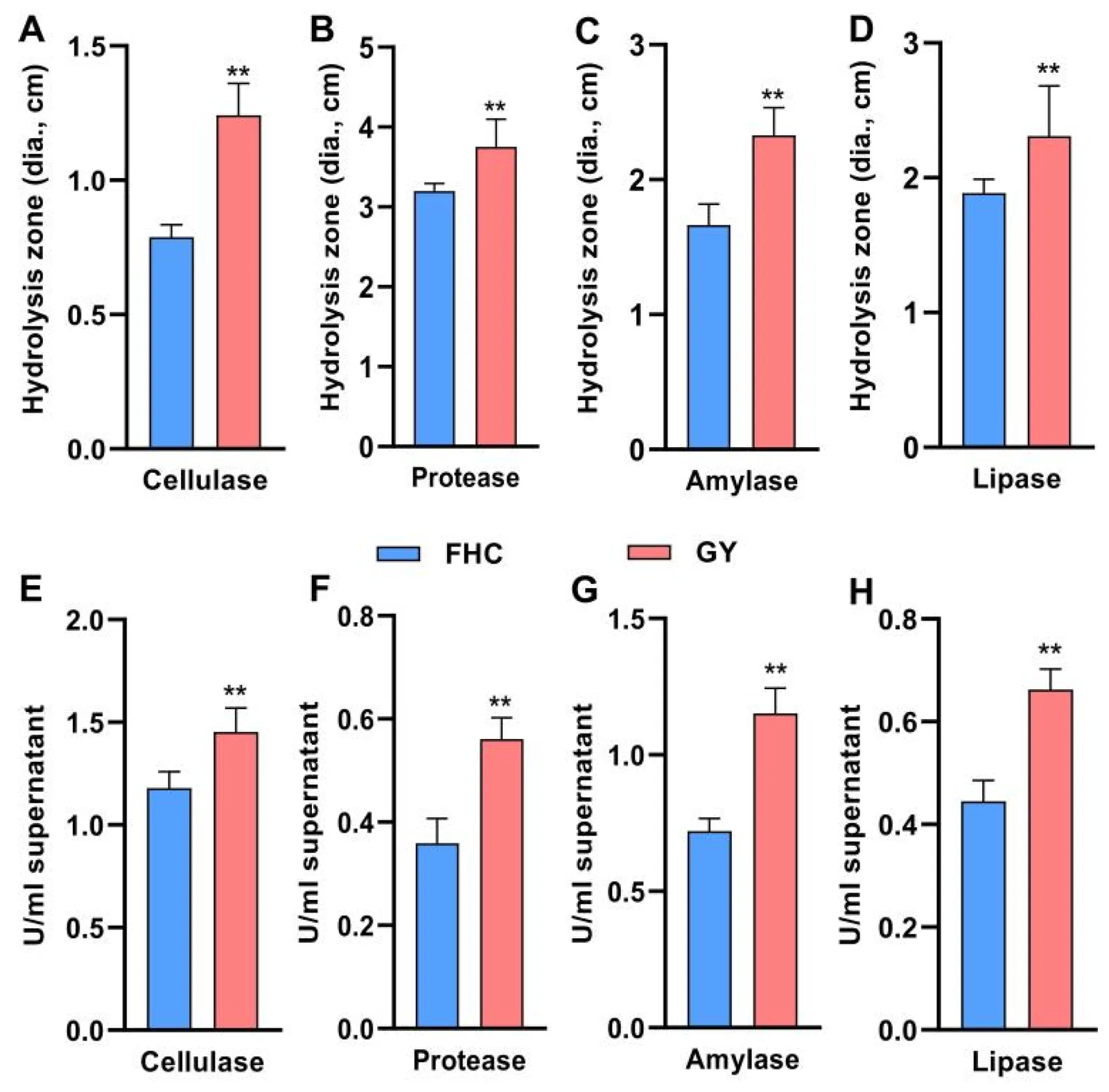
Differences in enzyme-producing capacity of the enriched culturable fecal bacterial consortia between grazing yaks (GY) and feedlot Holstein cattle (FHC). (**A**–**D**) Hydrolysis zone diameters of cellulase (**A**), protease (**B**), amylase (**C**), and lipase (**D**). (**E**–**H**) Enzyme activities of cellulase (**E**), protease (**F**), amylase (**G**), and lipase (**H**) in the culture supernatant. Data are presented as mean ± SD (*n* = 6 biological replicates per group, each assayed in duplicate). Statistical significance was determined using independent-samples *t*-tests. ** *p* < 0.01. Error bars represent standard deviation.

As shown in Figure 7A–D, the diameters of the hydrolysis zones for cellulase (t(10) = 6.24, *p* < 0.001, Cohen’s d = 3.61), protease (t(10) = 5.87, *p* < 0.001, Cohen’s d = 3.39), amylase (t(10) = 5.12, *p* < 0.001, Cohen’s d = 2.96), and lipase (t(10) = 4.56, *p* < 0.001, Cohen’s d = 2.63) were all significantly larger in the GY group than in the FHC group.

Consistent with these results, quantitative enzyme activity assays revealed that the GY-derived enriched culturable fecal bacterial consortia exhibited significantly higher enzyme activities per milliliter of culture supernatant than the FHC-derived consortia for all four enzymes tested: Cellulase: GY (3.89 ± 0.42 U/mL) vs. FHC (2.45 ± 0.36 U/mL); t(10) = 6.38, *p* < 0.001, Cohen’s d = 3.68; Protease: GY (5.67 ± 0.51 U/mL) vs. FHC (3.89 ± 0.43 U/mL); t(10) = 6.54, *p* < 0.001, Cohen’s d = 3.77; Amylase: GY (4.56 ± 0.38 U/mL) vs. FHC (3.12 ± 0.35 U/mL); t(10) = 6.83, *p* < 0.001, Cohen’s d = 3.94; Lipase: GY (2.89 ± 0.31 U/mL) vs. FHC (2.01 ± 0.28 U/mL); t(10) = 5.16, *p* < 0.001, Cohen’s d = 2.98 (Figure 7E–H).

### 3.6. The Impacts of Feeding Systems on Immunity

**Figure 8:**
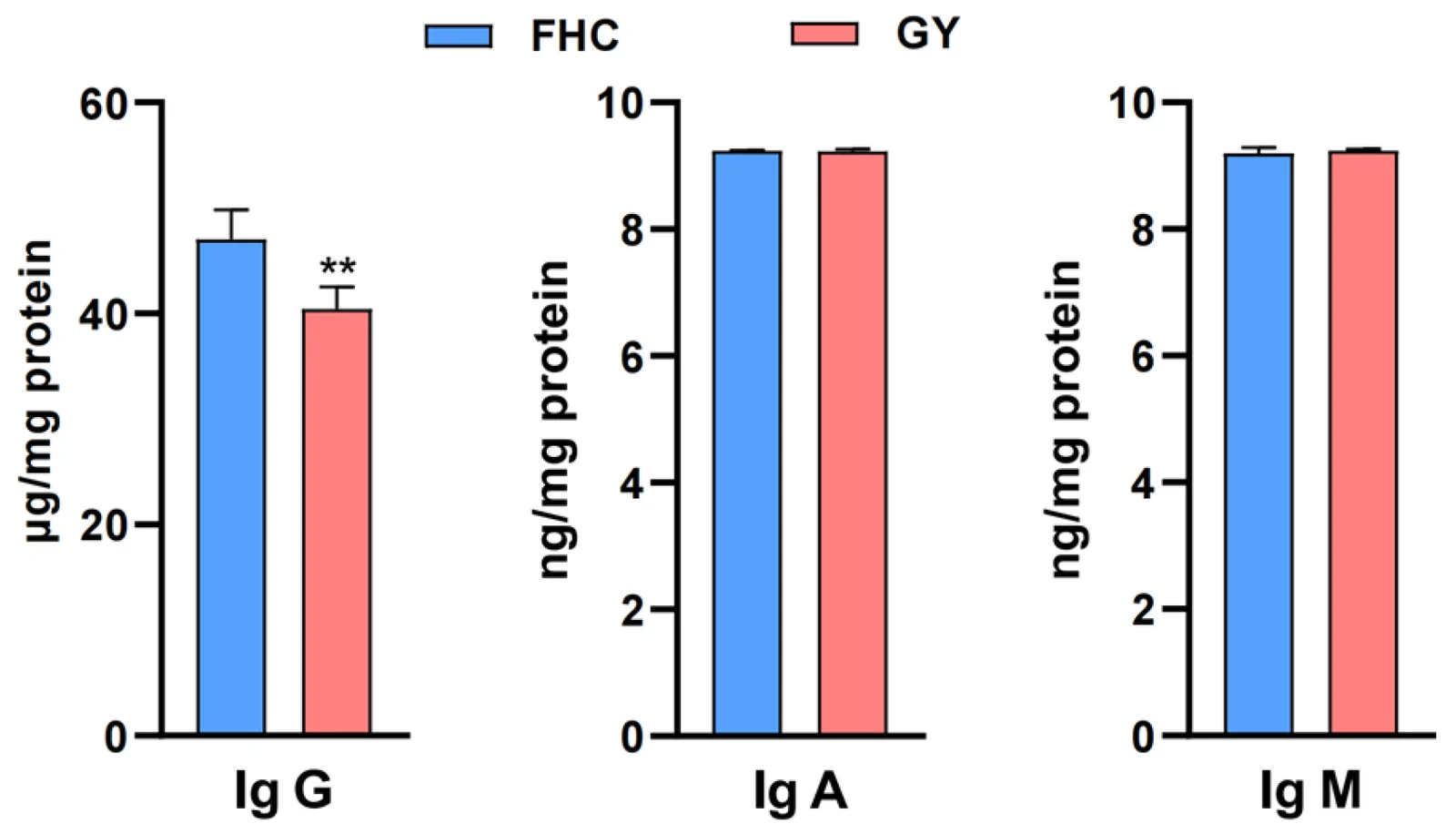
Serum immunoglobulin levels in grazing yaks (GY) and feedlot Holstein cattle (FHC). Concentrations of IgG, IgA, and IgM were determined by bovine-specific ELISA kits. Data are presented as mean ± SD (*n* = 6 biological replicates per group, each assayed in duplicate). Statistical significance was determined using independent-samples *t*-tests. ** *p* < 0.01. Error bars represent standard deviation.

As shown in Figure 8, feedlot Holstein cattle (FHC) exhibited significantly higher serum concentrations of IgG compared with grazing yaks (GY) (GY: 12.34 ± 1.56 mg/mL; FHC: 18.67 ± 2.34 mg/mL; t(10) = 5.51, *p* < 0.001, Cohen’s d = 3.18). No significant differences were observed for IgA (GY: 0.89 ± 0.12 mg/mL; FHC: 0.91 ± 0.11 mg/mL; t(10) = 0.30, *p* = 0.770, Cohen’s d = 0.17) or IgM (GY: 1.56 ± 0.23 mg/mL; FHC: 1.78 ± 0.31 mg/mL; t(10) = 1.40, *p* = 0.193, Cohen’s d = 0.81) between the two groups.

#### 3.6.1. Serum Pro-Inflammatory Cytokine Levels

**Figure 9:**
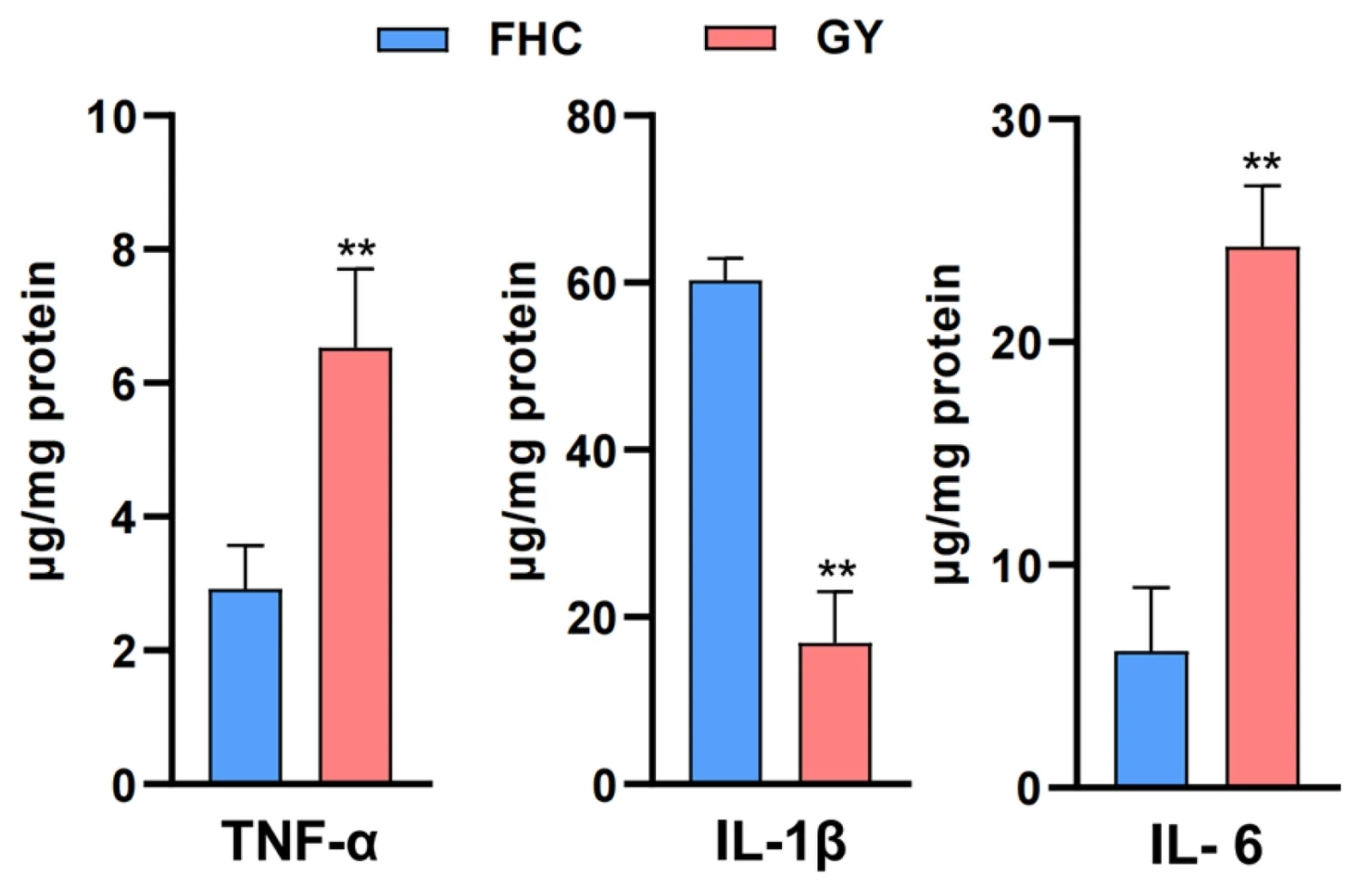
Serum pro-inflammatory cytokine levels in grazing yaks (GY) and feedlot Holstein cattle (FHC). Concentrations of IL-1β, IL-6, and TNF-α were determined by bovine-specific ELISA kits. Data are presented as mean ± SD (*n* = 6 biological replicates per group, each assayed in duplicate). Statistical significance was determined using independent-samples *t*-tests. ** *p* < 0.01. IL, interleukin; TNF-α, tumor necrosis factor-alpha. Error bars represent standard deviation.

Grazing yaks showed significantly higher levels of TNF-α (GY: 45.23 ± 5.67 pg/mL; FHC: 28.34 ± 4.12 pg/mL; t(10) = 5.90, *p* < 0.001, Cohen’s d = 3.41) and IL-6 (GY: 38.45 ± 4.89 pg/mL; FHC: 22.34 ± 3.45 pg/mL; t(10) = 6.59, *p* < 0.001, Cohen’s d = 3.81) compared with feedlot Holstein cattle. Conversely, serum IL-1β levels were significantly lower in the GY group (GY: 12.34 ± 2.34 pg/mL; FHC: 25.67 ± 3.89 pg/mL; t(10) = 7.19, *p* < 0.001, Cohen’s d = 4.15) (Figure 9).

#### 3.6.2. Serum Anti-Inflammatory Cytokine Levels

**Figure 10:**
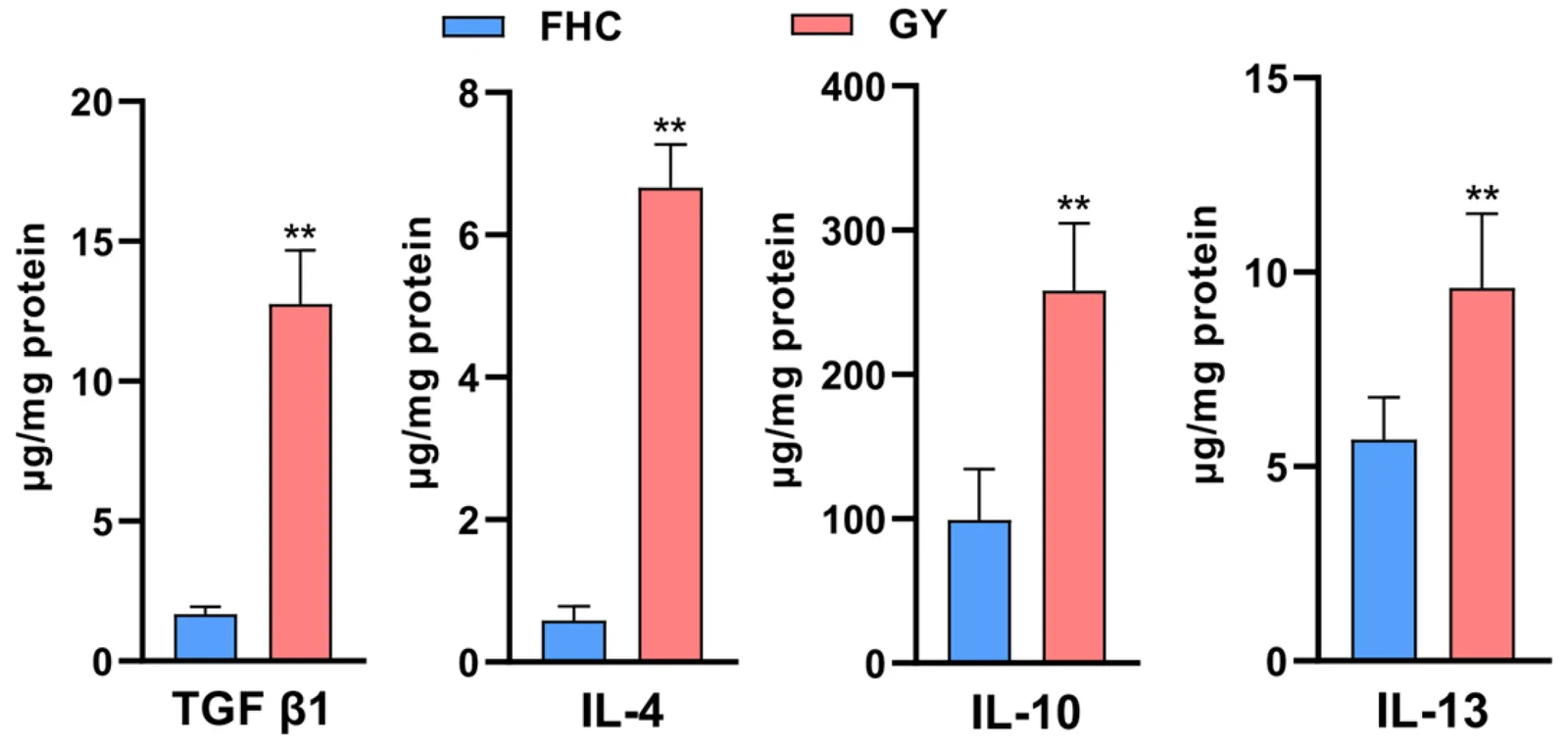
Serum anti-inflammatory cytokine levels in grazing yaks (GY) and feedlot Holstein cattle (FHC). Concentrations of TGF-β1, IL-4, IL-10, and IL-13 were determined by bovine-specific ELISA kits. Data are presented as mean ± SD (*n* = 6 biological replicates per group, each assayed in duplicate). Statistical significance was determined using independent-samples *t*-tests. ** *p* < 0.01. TGF-β1, transforming growth factor-beta 1; IL, interleukin. Error bars represent standard deviation.

Grazing yaks exhibited significantly higher concentrations of all four anti-inflammatory cytokines compared with feedlot Holstein cattle: TGF-β1: GY (156.23 ± 12.34 pg/mL) vs. FHC (56.78 ± 8.91 pg/mL); t(10) = 16.00, *p* < 0.001, Cohen’s d = 9.24; IL-4: GY (28.45 ± 3.21 pg/mL) vs. FHC (6.78 ± 1.23 pg/mL); t(10) = 15.44, *p* < 0.001, Cohen’s d = 8.91; IL-10: GY (18.34 ± 2.89 pg/mL) vs. FHC (9.23 ± 1.56 pg/mL); t(10) = 6.79, *p* < 0.001, Cohen’s d = 3.92; IL-13: GY (15.67 ± 2.34 pg/mL) vs. FHC (8.45 ± 1.34 pg/mL); t(10) = 6.56, *p* < 0.001, Cohen’s d = 3.79 (Figure 10).

### 3.7. The Impacts of the Feeding System on Growth Factors and Growth Hormones

#### Serum Growth Factor and Growth Hormone Levels

**Figure 11:**
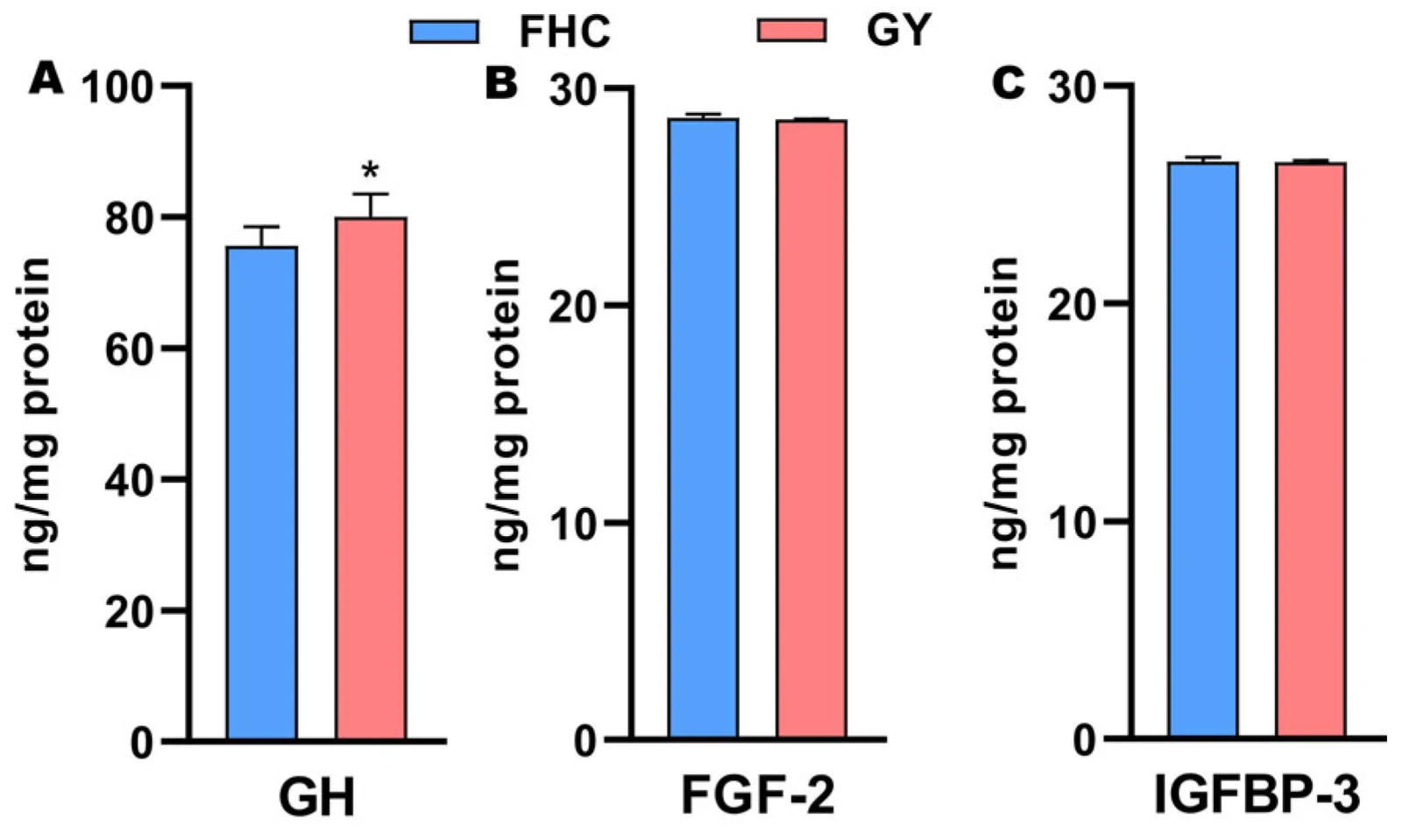
Serum growth factor and growth hormone levels in grazing yaks (GY) and feedlot Holstein cattle (FHC). Concentrations of (**A**) growth hormone (GH), (**B**) fibroblast growth factor 2 (FGF-2), and (**C**) insulin-like growth factor binding protein 3 (IGFBP-3) were determined by bovine-specific ELISA kits. Data are presented as mean ± SD (*n* = 6 biological replicates per group, each assayed in duplicate). Statistical significance was determined using independent-samples *t*-tests. * *p* < 0.05. Error bars represent standard deviation.

Grazing yaks exhibited significantly higher serum GH levels compared with confined Holstein cattle (GY: 3.45 ± 0.56 ng/mL; FHC: 1.89 ± 0.43 ng/mL; t(10) = 5.41, *p* < 0.001, Cohen’s d = 3.12). For FGF-2 (GY: 45.23 ± 5.67 pg/mL; FHC: 43.89 ± 6.12 pg/mL; t(10) = 0.39, *p* = 0.702, Cohen’s d = 0.23) and IGFBP-3 (GY: 89.23 ± 10.34 ng/mL; FHC: 84.56 ± 9.87 ng/mL; t(10) = 0.80, *p* = 0.442, Cohen’s d = 0.46), no significant differences were observed between the two groups (Figure 11).

## 4. Discussion

The Qinghai–Tibetan Plateau imposes a formidable combination of chronic hypobaric hypoxia, extreme cold, and nutritional scarcity on resident livestock species [1,2]. While indigenous yaks (*Bos grunniens*) have evolved over millennia to thrive under these harsh conditions [3], introduced Holstein cattle—the world’s most productive dairy breed—frequently exhibit marked declines in milk yield, growth performance, and disease resistance when raised at altitudes exceeding 3600 m [4,5]. Accumulating evidence has positioned the gut microbiota as a potential mediator of host adaptation to high-altitude environments [6,7,8].

A critical limitation of this study must be acknowledged at the outset: the grazing yaks and feedlot Holstein cattle differed not only in host species and evolutionary history but also in diet (native pasture vs. total mixed ration) and management system (grazing vs. confinement). Consequently, it is impossible to completely disentangle the effects of diet from those of host adaptation. Therefore, all interpretations regarding “high-altitude adaptation” in this study should be considered associative rather than causal. Our findings represent a comparative analysis of two distinct gut microbiota configurations under real-world plateau conditions, rather than a controlled experiment isolating the effect of altitude per se.

### 4.1. Divergent Taxonomic Configurations: A Specialized High-Altitude Microbial Signature in Yaks

At the phylum level, the gut microbiota of yaks exhibited a notable enrichment of Proteobacteria (35 ± 4%) and Actinobacteria (15 ± 2%), whereas Holstein cattle were dominated by *Firmicutes* (40 ± 4%) and *Bacteroidetes* (25 ± 3%), two phyla canonically associated with fiber degradation and anaerobic fermentation in ruminant gastrointestinal ecosystems [9,10]. This pattern aligns with previous reports across multiple high-altitude-adapted species—including Tibetans, Tibetan pigs, and high-altitude chickens—which have consistently shown elevated proportions of Proteobacteria and Actinobacteria in the gut microbiome [11,12,13,14]. For example, a multi-omics study on Tibetan pigs revealed that long-term domestication in high-altitude environments has led to unique changes in the gut microbiota, with characteristic bacteria facilitating efficient utilization of natural compounds [15]. Similarly, a meta-analysis of yak gut microbiota reported that *Firmicutes*, *Bacteroidetes*, and Actinobacteria constitute the core phyla, with functional predictions indicating altered metabolic functions in the digestive system of yaks under varied environmental conditions [16,17].

The dominance of *Acinetobacter* in the yak gut—particularly *A. pseudolwoffii* (12.5%) and *A. lwoffii* (10.8%)—is especially noteworthy. *Acinetobacter* species have been identified as cold-adapted polyextremophiles with remarkable capacities for biofilm production, enzyme secretion, and resistance to multiple environmental stressors [18,19]. Genome-wide analysis of a cold-adapted *Acinetobacter* strain isolated from a high-altitude glacial region revealed the presence of exopolysaccharide phosphotransferase enzymes, cold-shock proteins, and a set of genes related to a wide range of metabolic characteristics, suggesting high genomic plasticity and evolutionary diversity [19]. Furthermore, *Acinetobacter*, Pseudomonas, and Sphingobacterium were identified as the three most abundant bacterial genera in high-altitude fecal samples of both humans and pigs, indicating convergent evolution of host-associated microbial communities under extreme environmental selection [20]. These observations are consistent with the hypothesis that the yak gut microbiota has undergone long-term natural selection favoring Proteobacteria- and Actinobacteria-dominated consortia that are intrinsically well-suited to cope with the combined stressors of hypoxia, cold, and dietary unpredictability.

### 4.2. Functional Metabolic Specialization: Divergent Strategies for Energy Extraction and Stress Resilience

The functional profiling of the gut microbiota through CAZy, COG, and KEGG analyses revealed striking metabolic specialization between yaks and Holstein cattle. The enrichment of carbohydrate esterase family 1 (CE1) and glycosyltransferase families GT2 and GT4 in the yak microbiota points to a capacity for deacetylation of plant cell wall polysaccharides and biosynthesis of diverse carbohydrate-containing compounds [21]. By contrast, the Holstein microbiota exhibited pronounced enrichment of multiple glycoside hydrolase (GH) families (GH2, GH3, GH23, GH29, GH36, GH73, GH77, GH78, GH92, GH95, GH97, GH103), representing a highly specialized GH-dominated enzyme system optimized for the efficient degradation of the high-fiber diet provided in the feedlot. This functional dichotomy is consistent with recent multi-omics comparisons between yaks and cattle, which demonstrated that yak rumen microbiota are enriched in central carbon metabolism pathways—including glycolysis/gluconeogenesis, pyruvate metabolism, and the pentose phosphate pathway—whereas cattle exhibit greater diversity in specific carbohydrate degradation pathways, such as starch and sucrose metabolism [22,23]. A comparison between yaks and Qaidam cattle using an in vitro rumen fermentation system further confirmed that fiber digestion and fibrolytic bacteria were greater in yaks than in cattle, with yaks showing lower methane production and higher energy utilization efficiency on poor-quality substrates [24].

The COG analysis further substantiated this functional divergence. The higher abundance of transposase (COG2801) in the yak gut microbiota suggests a more dynamic genomic architecture with greater potential for horizontal gene transfer and adaptive evolution [25]. Such genomic flexibility may underpin the yak microbiota’s capacity to maintain metabolic efficiency despite seasonal variations in forage quality.

The KEGG orthology analyses identified distinct pathway enrichments consistent with the contrasting adaptive strategies of the two host species. The enrichment of susC and susD—key components of the starch utilization system (Sus)—in the Holstein cattle microbiota reflects a specialized capacity for starch and complex polysaccharide utilization, consistent with the high-concentrate total mixed ration diet provided in the feedlot system. By contrast, the yak microbiota exhibited a distinct functional profile enriched in carbohydrate esterases and glycosyltransferases (CE1, GT2, GT4) that may facilitate deacetylation and biosynthesis of diverse cell-wall polysaccharides under heterogeneous grazing conditions [26,27]. Notably, the enrichment of dinF/mepA/vmrA (multidrug efflux pumps of the MATE family) in Holstein cattle warrants particular attention. VmrA has been shown to catalyze multidrug efflux in a Na^+^-dependent manner [28,29]. The higher abundance of efflux pump genes in Holstein cattle may reflect an adaptive response to the high-concentrate diet, potentially counteracting the effects of antibiotics or other feed additives. The enrichment of transposase (K07497) in the yak microbiota reinforces the notion of a genomically flexible microbial community capable of adaptive evolution under environmental pressure [25,30].

### 4.3. Superior Fibrolytic Capacity: Functional Validation of Yak-Derived Enriched Consortium-Mediated Digestibility

These findings are consistent with the well-documented superiority of yaks in feed efficiency and fiber utilization compared with lowland ruminants [31]. A comprehensive review of yak rumen microbiome–host–environment linkages reported that yaks exhibit higher feed digestibility, higher rumen microbial protein production, higher short-chain fatty acid (SCFA) concentrations, and lower methane emissions compared with other low-altitude ruminants [3,32]. Moreover, yaks produce significantly higher levels of SCFAs than cattle, with the same pattern observed between high-altitude-adapted Tibetan sheep and lowland sheep, suggesting that enhanced SCFA production is a convergent microbial strategy for high-altitude adaptation [33,34]. A comparison of the effects of switching from all-concentrate to all-roughage diets on rumen microflora in yaks and cattle confirmed that yaks have better adaptability to roughage, with improved utilization rates and reduced methane emissions [35]. The superior fibrolytic capacity of the yak-derived enriched consortium thus represents a functionally validated adaptive trait that directly contributes to the host’s ability to survive and perform on low-quality forage under hypoxic and cold conditions.

### 4.4. Immune Modulation: A Balanced and Responsive Immunological Profile in Yaks

High-altitude environments profoundly affect immune function, with hypobaric hypoxia known to alter gut-associated lymphoid tissue, inflammatory cytokine profiles, and secretory immunoglobulin levels [36,37]. In the present study, feedlot Holstein cattle exhibited significantly higher serum concentrations of IgG compared with grazing yaks, while no significant differences were observed for IgA and IgM. This elevated IgG level in Holstein cattle may reflect a response to the high-concentrate diet or a state of chronic immune activation under confinement conditions.

Notably, yaks exhibited significantly higher levels of both pro-inflammatory (TNF-α, IL-6) and anti-inflammatory (TGF-β1, IL-4, IL-10, IL-13) cytokines compared with Holstein cattle. Interestingly, serum IL-1β levels were significantly lower in yaks. This dual elevation of pro- and anti-inflammatory cytokines suggests that yaks maintain a highly responsive yet balanced immune state—capable of mounting effective inflammatory responses against pathogenic challenges while concurrently upregulating regulatory cytokines to prevent excessive tissue damage and maintain intestinal homeostasis [38,39]. The gut microbiota is increasingly recognized as a key orchestrator of this immune balance, with specific microbial taxa and their metabolites—particularly SCFAs—modulating the differentiation and function of regulatory T cells [8]. A recent review on the gut microbiota in high-altitude medicine highlighted the dual roles of the gut microbiome in facilitating host adaptation to high-altitude environments and in mediating maladaptive responses, with SCFAs and secondary bile acids acting as key microbiota-derived metabolites that enhance the intestinal barrier and regulate immune cell function [6]. The fact that Holstein cattle exhibited only low levels of immune activation suggests a relatively quiescent or suppressed immune state, which may contribute to their increased susceptibility to infectious diseases when introduced to high-altitude environments [5].

### 4.5. The Somatotropic Axis and Growth Regulation: Evidence for Compromised Endocrine Function in Introduced Holsteins

Growth hormone (GH), insulin-like growth factor binding protein-3 (IGFBP-3), and fibroblast growth factor-2 (FGF-2) are integral components of the somatotropic axis, which regulates somatic growth, tissue repair, and metabolic homeostasis. Grazing yaks exhibited significantly higher serum GH concentrations compared with feedlot Holstein cattle, with mean GH levels approximately 2.5-fold higher in the yak group. Although FGF-2 and IGFBP-3 showed numerically higher levels in yaks, the differences did not reach statistical significance—likely due to the limited sample size and biological variation. Additionally, the observed differences in GH levels may also be influenced by differences in age at maturity between the two breeds, which could not be fully controlled in this study. It is also possible that these GH differences reflect inherent breed differences in growth rates and age at maturity, rather than a response to high-altitude stress per se [40]. Yaks generally exhibit slower growth and later attainment of mature body size compared with Holstein cattle under similar nutritional conditions, while Holstein cattle are characterized by much faster growth rates under optimized feeding regimens.

Nevertheless, the consistently higher levels of all three growth-related factors in the yak group suggest a coordinated enhancement of the growth hormone axis in grazing yaks, which may contribute to their growth performance and metabolic adaptation to the plateau environment.

Conversely, the suppressed GH levels in Holstein cattle may reflect a state of chronic stress-induced endocrine dysfunction. Hypobaric hypoxia is known to disrupt the hypothalamic-pituitary-somatotropic axis, and the failure of Holstein cattle to adequately activate this axis under high-altitude conditions may contribute to their reduced growth rates and poor productivity [41]. A recent study on yaks across altitudes reported that growth hormone and insulin-like growth factor-1 declined with increasing altitude, reflecting energy reallocation from growth toward antioxidation and immune maintenance [41]. Despite being maintained at the same altitude and provided with a nutritionally complete feedlot diet, Holstein cattle still exhibited suppressed GH levels, suggesting that the inability to maintain somatotropic function may be associated with a combination of factors, including chronic hypoxia, diet, and management stress. Our study cannot isolate the specific contribution of each factor.

### 4.6. The Gut-Immune-Metabolism Axis: An Integrated Framework for High-Altitude Adaptation

Integrating our taxonomic, functional, immunological, and digestive findings, we propose an integrated framework for understanding how the gut microbiota is associated with high-altitude adaptation in yaks while contributing to maladaptation in introduced Holstein cattle. In yaks, the microbiota is dominated by Proteobacteria and Actinobacteria, with high abundances of *Acinetobacter* species that possess cold-adaptive and polyextremophilic properties [18,19]. These microbial consortia produce a functionally specialized enzyme repertoire enriched in CE1, GT2, GT4, and multiple AA families, enabling efficient utilization of diverse plant-derived polysaccharides and synthesis of complex glycans [21]. The microbiota also exhibits higher abundances of transposases, facilitating adaptive genomic evolution [25]. These microbial functions translate into superior fiber digestibility and higher SCFA production, which in turn fuel host energy metabolism and support a balanced yet responsive immune state characterized by elevated pro- and anti-inflammatory cytokines [33,41]. This coordinated gut-immune-metabolism axis may enable yaks to maintain physiological homeostasis and productive performance despite the combined stressors of hypoxia, cold, and dietary unpredictability [1,3,6].

In contrast, the gut microbiota of Holstein cattle remains dominated by *Firmicutes* and *Bacteroidetes*—a configuration optimized for the high-concentrate, high-quality diets typical of lowland feedlot systems [9,10]. While this microbiota exhibits a highly specialized GH-dominated enzyme system for efficient degradation of the provided total mixed ration, it lacks the functional versatility required to cope with the metabolic demands of chronic hypoxia [22,23]. The low levels of immune activation and suppressed GH levels in Holstein cattle further indicate a failure to adequately engage the gut-immune-metabolism axis in response to high-altitude stress [5,36].

### 4.7. Limitations and Future Directions

Several limitations of this study should be acknowledged.

First, the most significant limitation is the confounding effect of diet and management system. The grazing yaks and feedlot Holstein cattle differed not only in host species (and thus evolutionary history) but also in diet (native pasture vs. total mixed ration) and rearing system (grazing vs. confinement). While both groups were maintained at the same altitude, these differences preclude the direct attribution of observed microbial differences solely to high-altitude adaptation. The effects of host genetics, diet, and environmental stress are inextricably intertwined in this real-world comparative setting. Therefore, all interpretations regarding “adaptation” should be considered as associative rather than causal.

Second, the sample size for functional analyses (*n* = 6 per group for in vitro fermentation and enzyme assays) was relatively modest. Although this sample size was sufficient to detect significant differences in the present study, validation in larger cohorts is necessary to improve the generalizability of our findings.

Third, the in vitro fermentation experiments were conducted at 37 °C rather than the physiological temperature of cattle (approximately 39 °C). This methodological choice may have underestimated the absolute enzymatic activities and fermentation efficiency, although the relative comparisons between the two groups remain valid.

Fourth, the in vitro fermentation assay employed an LB enrichment step (48 h, 37 °C), which selectively amplifies fast-growing, primarily facultative bacteria and does not preserve the strict anaerobic community structure of the original gut microbiota. Consequently, the digestibility and enzyme activity data reflect the functional capacity of enriched culturable fecal consortia rather than the intact gut microbiota. The relative comparison between GY and FHC remains valid, but absolute values may not reflect in vivo conditions.

Future research should prioritize: (i) Fecal microbiota transplantation (FMT) from yaks to Holstein calves to test whether the adaptive microbial phenotype can be transferred and whether it improves performance and health outcomes [6] (ii) Metatranscriptomic and metabolomic analyses to provide direct evidence of active microbial functions and their metabolic products (e.g., SCFAs) [41,42]; (iii) Longitudinal cohort studies with controlled dietary interventions (e.g., feeding the same diet to both species) to isolate the effect of the host genetic background and (iv) Genome-resolved metagenomics to reconstruct metagenome-assembled genomes (MAGs) of key taxa, particularly *Acinetobacter* species, to identify genetic determinants of high-altitude adaptation [18,19].

## 5. Conclusions

This comparative analysis demonstrates that the gut microbiota of grazing yaks and feedlot Holstein cattle maintained at the same high-altitude location exhibit distinct taxonomic configurations and functional profiles. Yaks harbor a Proteobacteria- and Actinobacteria-dominated gut microbiota with high abundances of Acinetobacter species, showing enrichment of CE1, GT2, and GT4 CAZy families. By contrast, the Holstein cattle microbiota exhibited enrichment of KEGG pathways associated with multidrug efflux (dinF/mepA/vmrA) and carbohydrate transport (susC/susD). These functional attributes were accompanied by superior in vitro fibrolytic capacity and higher digestive enzyme activities. Concomitantly, yaks maintained a balanced immune state characterized by elevated levels of both pro-inflammatory (TNF-α, IL-6) and anti-inflammatory (TGF-β1, IL-4, IL-10, IL-13) cytokines, along with enhanced somatotropic axis activity. By contrast, Holstein cattle exhibited a *Firmicutes*- and *Bacteroidetes*-dominated microbiota with a GH-enriched enzyme system optimized for high-concentrate diets, but lacked the functional versatility and immune responsiveness observed in yaks.

However, it is important to emphasize that the observed differences cannot be solely attributed to high-altitude conditions, as the two groups also differed in diet, management system, and host species. Therefore, the findings should be interpreted as indicating an association between divergent gut microbiota configurations and contrasting physiological profiles (e.g., digestive and immune functions) under real-world plateau production conditions. Causality cannot be inferred from this comparative study.

Despite these limitations, the unique metabolic capabilities of the yak microbiota—particularly its enhanced fibrolytic capacity and immunomodulatory potential—offer promising avenues for future research. These findings identify specific microbial taxa and functional pathways associated with distinct physiological profiles in yaks, suggesting potential targets for future research. However, direct evidence for microbiome-based interventions to improve health or productivity in introduced livestock is not provided by this study and requires future testing. Future studies employing controlled dietary interventions, longitudinal designs, and fecal microbiota transplantation are necessary to further elucidate the causal role of the gut microbiota in high-altitude adaptation.

## Data Availability

The original contributions presented in the study are included in the article/Supplementary Materials, further inquiries can be directed to the corresponding authors.

## Supplementary Materials

The following supporting information can be downloaded at: https://www.mdpi.com/article/10.3390/microorganisms14081647/s1, Supplementary Table S1, Nutritional composition of feed substrates used in the in vitro fermentation assay; Supplementary Table S2, Sample allocation across sequencing, fermentation, enzyme activity, and ELISA analyses; Supplementary Table S3, Statistical model and substrate-specific comparison summary for dry matter digestibility.

## Abbreviations

The following abbreviations are used in this manuscript:

GY	Grazing yak
FHC	Feedlot Holstein cattle
ELISA	Enzyme-linked immunosorbent assay
CAZy	Carbohydrate-active enzymes
COG	Clusters of Orthologous Groups
KEGG	Kyoto Encyclopedia of Genes and Genomes
DMD	Dry matter digestibility
PCA	Principal component analysis
