# Supplementary material for: Divergent Gut Microbiota Configurations Are Associated with Contrasting Physiological Profiles in Grazing Yaks and Introduced Feedlot Holstein Cattle Under High-Altitude Conditions
Source: Microorganisms. 2026 Jul 28;14(8):1647. doi: 10.3390/microorganisms14081647 (PMC13515109; doi:10.3390/microorganisms14081647)
Supplement: Supplementary file 1 [file microorganisms-14-01647-s001.zip › microorganisms-4445081-supplementary.pdf]

Supplementary Table S1. Nutritional composition of six feed substrates used in the in vitro fermentation assay (%, dry matter basis)..

Substrate type	Crude protein (%)	Neutral detergent fiber (%)	Acid detergent fiber (%)	Source
Corn straw	5.0	68.0	40.0	China Feed Database (2024)
Wheat straw	3.5	75.0	48.0	China Feed Database (2024)
Oat straw	5.5	70.0	42.0	FAO (2023)
Barley straw	4.2	72.5	45.3	China Feed Database (2024)
Rice straw	4.0	70.0	44.0	FAO (2023)
Basic diet	30.0	21.0	24.0	Laboratory analysis

Note: The nutritional composition data for the straw substrates were obtained from the China Feed Database (2024) and the FAO Grassland and Pasture Crops Database (2023). The values for the concentrate mixture were obtained from the manufacturer's specification (or laboratory analysis). These values represent the typical nutritional composition of feedstuffs commonly used in the Tibetan Plateau region.

Supplementary Table S2. Sample allocation across analyses

Analysis	Sample type	GY biological replicates	FHC biological replicates	Technical replicates	Notes
16S rRNA amplicon sequencing	Feces	20	20	No	Individual fecal samples; no pooling
Shotgun metagenomic sequencing	Feces	20	20	No	Same DNA samples as used for 16S sequencing
CAZy/COG/KEGG profiling	Shotgun metagenomic data	20	20	No	Based on individual metagenomes
Alpha diversity	16S/metagenomic data	20	20	No	Individual fecal samples
Beta diversity	16S/metagenomic data	20	20	No	Individual fecal samples
In vitro fermentation/DMD	Enriched fecal bacterial consortium	6	6	Yes	Technical replicates were averaged
Enzyme activity assays	Culture supernatant	6	6	Yes	Technical replicates were averaged
ELISA	Serum	6	6	Duplicate wells	Individual serum samples

Supplementary Table S3A. Statistical model and correction strategy

Item	Description
Response variable	Dry matter digestibility (%)
Biological replicate	Individual animal-derived enriched culturable fecal bacterial consortium
Biological replicates	GY, n = 6; FHC, n = 6
Technical replicates	Triplicate measurements for each substrate; technical replicates were averaged before statistical analysis
Fixed factors	Bacterial origin, substrate type, and bacterial origin × substrate type interaction
Overall test	Two-way ANOVA
Substrate-specific comparisons	Independent-samples t-test comparing GY and FHC within each substrate
Multiple-comparison correction	Bonferroni correction for six substrate-specific comparisons
Adjusted significance threshold	$\alpha = 0.0083$ (0.05/6)
Effect sizes	Partial η^2 for ANOVA; Cohen's d for pairwise comparisons
Software	SPSS 26.0
Interpretation	A substrate-specific difference was considered significant only when the unadjusted P value was below 0.0083, equivalent to a Bonferroni-adjusted P value below 0.05

Supplementary Table S3B. Two-way ANOVA results for dry matter digestibility

Effect	df1	df2	F	P	Partial η^2
Bacterial origin	1	10	48.21	<0.001	0.83
Substrate type	5	50	12.35	<0.001	0.55
Bacterial origin × substrate type	5	50	3.57	0.008	0.26

Partial η^2 values of 0.01, 0.06, and 0.14 were interpreted as small, medium, and large effects, respectively.

Supplementary Table S3C. Substrate-specific descriptive statistics

Substrate	GY, mean ± SD (%)	FHC, mean ± SD (%)	Mean difference, GY − FHC (95% CI)
Corn straw	45.32 ± 3.21	38.15 ± 2.87	7.17 (3.25 to 11.09)
Wheat straw	42.18 ± 3.45	36.54 ± 2.92	5.64 (1.53 to 9.75)
Oat straw	40.56 ± 3.12	35.22 ± 2.68	5.34 (1.60 to 9.08)
Barley straw	47.89 ± 3.56	39.67 ± 2.45	8.22 (4.29 to 12.15)
Rice straw	43.21 ± 3.33	36.89 ± 2.76	6.32 (2.39 to 10.25)
Concentrate mixture (Basic diet)	51.67 ± 2.98	46.23 ± 2.54	5.44 (1.88 to 9.00)

Supplementary Table S3D. Substrate-specific pairwise comparisons

Substrate	t (df = 10)	Unadjusted P	Bonferroni-adjusted P	Cohen's d	Interpretation
Corn straw	4.08	0.002	0.013	2.35	Significant
Wheat straw	3.06	0.012	0.073	1.76	Not significant
Oat straw	3.18	0.010	0.059	1.84	Not significant
Barley straw	4.66	<0.001	0.005	2.69	Significant
Rice straw	3.58	0.005	0.030	2.07	Significant
Concentrate mixture (Basic diet)	3.40	0.007	0.040	1.96	Significant

Two-sided unadjusted P values were obtained from independent-samples t-tests. Bonferroni-adjusted P values were calculated as min(6 × unadjusted P, 1.0). Statistical significance after correction was defined as an unadjusted $P < 0.0083$ or, equivalently, a Bonferroni-adjusted $P < 0.05$. Cohen's d values of 0.2, 0.5, and 0.8 were interpreted as small, medium, and large effects, respectively.
